# Targeted synthesis of a trimethoxyphenyltetrahydropyrimidine analogue designed as a DNA intercalator: *in silico*, multi-spectroscopic, thermodynamic, and *in vitro* approaches†

**DOI:** 10.1039/d5ra02179k

**Published:** 2025-05-08

**Authors:** Ahmed A. Al-Karmalawy, Ayman Abo Elmaaty, Galal Magdy, Aya Saad Radwan, Radwan Alnajjar, Moataz A. Shaldam, Arwa Omar Al Khatib, Salem Salman Almujri, Abdullah Yahya Abdullah Alzahrani, Haytham O. Tawfik

**Affiliations:** a Department of Pharmaceutical Chemistry, College of Pharmacy, The University of Mashreq Baghdad 10023 Iraq akarmalawy@horus.edu.eg; b Department of Pharmaceutical Chemistry, Faculty of Pharmacy, Horus University-Egypt New Damietta 34518 Egypt; c Medicinal Chemistry Department, Faculty of Pharmacy, Port Said University Port Said 42526 Egypt; d Medicinal Chemistry Department, Clinical Pharmacy Program, East Port Said National University Port Said 42526 Egypt; e Pharmaceutical Analytical Chemistry Department, Faculty of Pharmacy, Kafrelsheikh University Kafrelsheikh 33511 Egypt; f Department of Pharmaceutical Analytical Chemistry, Faculty of Pharmacy, Mansoura National University Gamasa 7731168 Egypt; g CADD Unit, Faculty of Pharmacy, Libyan International Medical University Benghazi 16063 Libya; h Department of Pharmaceutical Chemistry, Faculty of Pharmacy, Kafrelsheikh University Kafrelsheikh 33516 Egypt; i Faculty of Pharmacy, Hourani Center for Applied Scientific Research, Al-Ahliyya Amman University Amman Jordan; j Department of Pharmacology, College of Pharmacy, King Khalid University Asir-Abha 61421 Saudi Arabia; k Department of Chemistry, Faculty of Science, King Khalid University Abha 61413 Saudi Arabia; l Department of Pharmaceutical Chemistry, Faculty of Pharmacy, Tanta University Tanta 31527 Egypt haytham.omar.mahmoud@pharm.tanta.edu.eg

## Abstract

Based on the rational design of DNA intercalators and Topo-II inhibitors and taking into consideration the main pharmacophoric features of doxorubicin (Dox) as a reference standard, we theoretically designed novel substituted tetrahydropyrimidine analogues (T_1–35_). The designed analogues (T_1–35_) were investigated for their inhibitory potential towards the hybrid DNA and Topo-II target receptor using molecular docking. Interestingly, the theoretically designed analogue T_30_ with a 3,4,5-trimethoxy phenyl side chain was found to be the superior candidate, achieving a binding score of −7.06 kcal mol^−1^, compared with two reference standards, doxorubicin (Dox) and a co-crystal ligand (EVP). Moreover, the docked candidates (T_30_, Dox, and EVP) were further subjected to molecular dynamics simulations for 500 ns. Furthermore, MM-GBSA calculations showed that the target candidate (T_30_) achieved superior Δ*G* binding energy (−33.86 kcal mol^−1^) compared with Dox and EVP. Moreover, T_30_ was found to be the most promising candidate that could be conveniently synthesized based on its order in the chemical synthesis scheme. In addition, to evaluate the antiproliferative activity and scope of compound T_30_, we requested the National Cancer Institute (NCI) to test it against nine cancer cell types. Interestingly, compound T_30_ exhibited very strong antiproliferative activity with a mean GI% of 122% and a mean GI_50_ of 4.10 μM. It exhibited the highest anticancer activity towards all 59 cell lines. Moreover, the *in vitro* binding interaction of compound T_30_ with calf thymus DNA (ctDNA) was examined using various techniques, such as spectrofluorimetry, UV-vis spectrophotometry, viscosity measurements, ionic strength measurements, and thermodynamics to confirm its mechanism of action. Investigating the intermolecular binding interaction between small compounds and DNA can provide valuable insights for designing drugs with enhanced effectiveness and improved targeted activities.

## Abstract 2

Based on the rational design of DNA intercalators and Topo-II inhibitors like doxorubicin (Dox) as a reference standard; ***methoxyphenyltetrahydropyrimidine a***a trimethoxyphenyltetrahydropyrimidine analogue was designed as a DNA intercalator.

## Graphical abstract

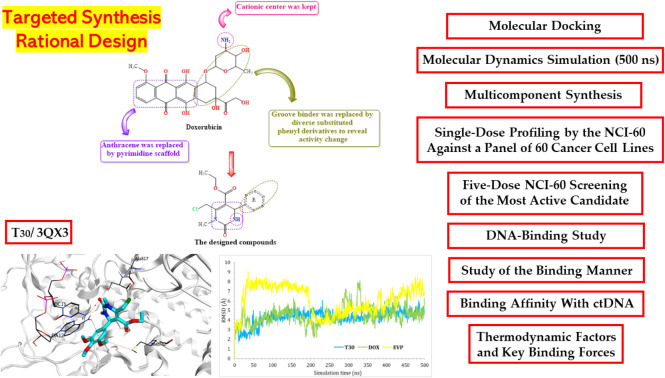

## 1. Introduction

Cancer remains a leading global health challenge, claiming approximately 8 million lives annually. Alarmingly, projections suggest a rise of over 50% in new cancer cases in the coming years.^1^ By 2030, the annual global burden of cancer is expected to reach a staggering 22 million cases.^2^ This represents a significant increase from the current 8 million cases per year, underlining the urgent need for advancements in cancer prevention, diagnosis, and treatment. A growing concern in cancer treatment is the emergence of acquired resistance to chemotherapy in various cancer types.^3,4^ Unfortunately, many chemotherapeutic drugs can also affect healthy dividing cells, causing side effects such as nausea, a weakened immune system, anemia, and hair loss. This is because these drugs are not specific in targeting only cancer cells.^5,6^

**Fig. 1:**
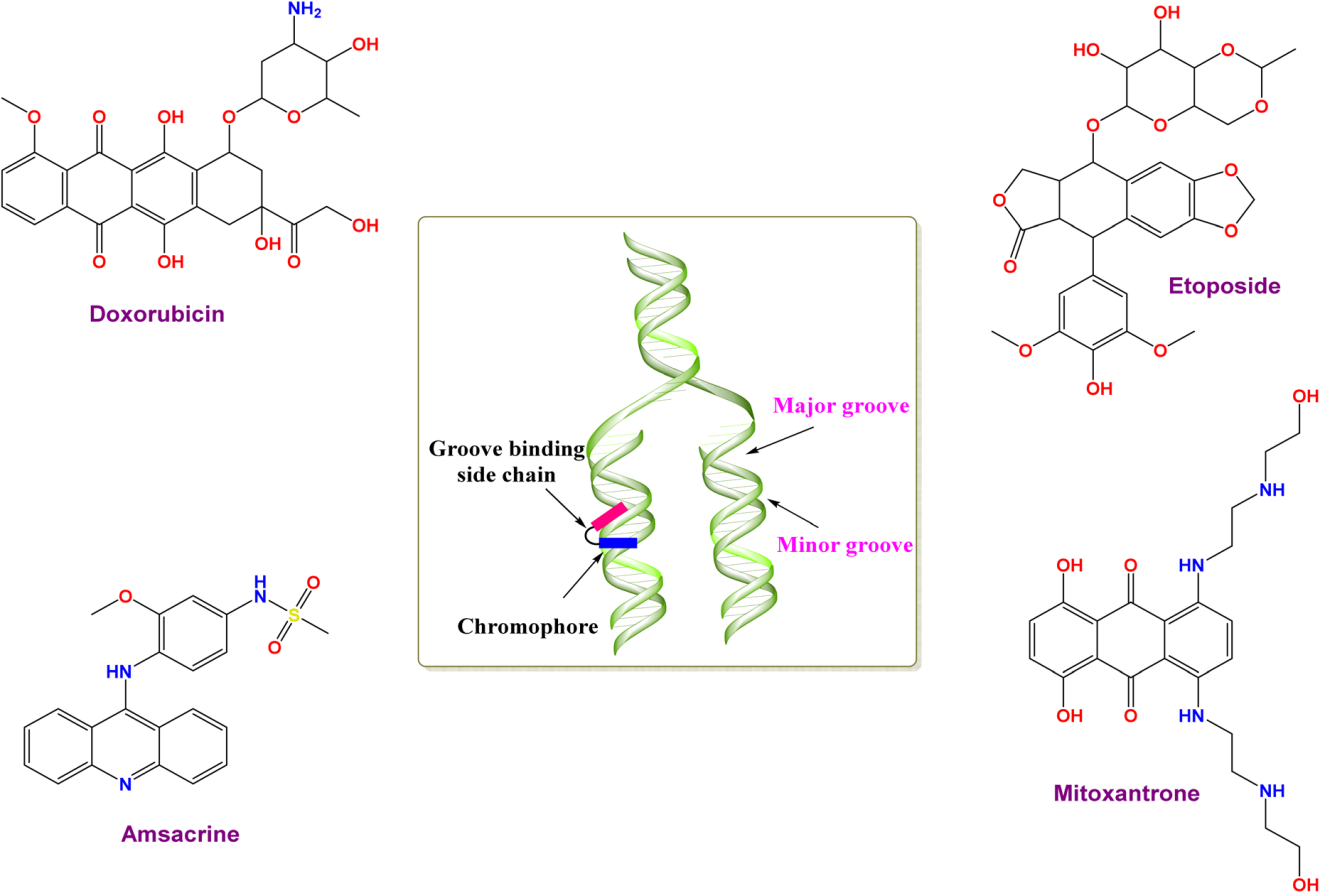
Illustration of some approved topoisomerase II (Topo-II) inhibitors and DNA intercalators.

This critical need for improved cancer treatments underscores the urgency of developing new anticancer agents with a superior therapeutic index. Many successful anticancer drugs work by causing DNA damage in cancer cells, ultimately leading to programmed cell death (apoptosis).^7,8^ Some anticancer drugs, called DNA intercalators, work by embedding themselves between the strands of DNA. This disrupts the normal function of DNA, leading to cell cycle disruptions and eventually cell death.^9,10^ By imagining the DNA molecule as a twisted ladder, DNA intercalators are similar to tiny wedges that can squeeze between the rungs of the ladder (base pairs). These wedges disrupt the normal functioning of the DNA, ultimately leading to cell death. Intercalation is stabilized by a combination of forces, including π-electron interactions, electrostatic attractions, and hydrophobic/polar interactions.^11^ Acridine derivatives (*e.g.* amsacrine),^12^ anthracyclines (*e.g.*, mitoxantrone, doxorubicin (Dox), and nogalamycin),^13,14^ are examples of FDA-approved DNA intercalators, as shown in Fig. 1.

Compounds can interact with DNA through either non-covalent or covalent interactions, with the non-covalent pattern typically being more prevalent. Non-covalent binding can be categorized into three types: intercalation, electrostatic, and groove binding.^15^ The molecule can undergo binding at several sites, including the minor groove and major groove, between two base pairs (full intercalation), on the exterior of the helix, and through electrostatic binding.^15,16^ Typically, there is no singular method that can offer a definitive understanding of the interaction between drugs and DNA. Therefore, it is crucial to provide effective, rapid, and economical experimental and computational methods to evaluate the interaction between DNA and other substances, with the purpose of assisting in drug design and discovery.

DNA topoisomerases are like tiny cellular machines (enzymes) that help manage the complex structure of DNA. DNA topoisomerases use energy from ATP to manipulate the molecular motion of DNA, preventing it from getting tangled.^17^ There are two main types of DNA topoisomerases: Type I and Type II. Notably, Type I enzymes (topoisomerase I) can make changes to the structure of a single strand of DNA, while Type II (topoisomerase II) enzymes can modify the structure involving both strands of DNA.^17^

Topoisomerase II (Topo-II) is regarded as a prominent enzyme in our cells that helps manage the complex structure of DNA. It temporarily aids in cutting both strands of the DNA molecule and then sealing the cuts back up. This allows DNA to unwind and be used in important cellular processes like copying chromosomes, cell division, and making proteins.^18^ Topoisomerase II inhibitors can be categorized into two major classes: Topo-II poisons and Topo-II catalytic inhibitors. Topo-II poisons are characterized by their ability to enhance the rate of Topo-II/DNA complex cleavage, ultimately leading to the formation of DNA strand lesions.^19^ Examples of Topo-II poisons are anthracyclines (*e.g.*, Dox^13^ and mitoxantrone^14^). However, Topo-II catalytic inhibitors work by specifically inhibiting the catalytic activity of Topo-II and do not change the rate of Topo-II/DNA complex cleavage.^20^ An example of a Topo-II catalytic inhibitor is Etoposide.^21^ Hence, DNA intercalation and Topo-II inhibition are emerging as attractive and efficient tools in the fight against cancer. Their ability to target DNA makes them valuable approaches for cancer therapy.

Additionally, the pyrimidine derivatives are privileged scaffolds in medicinal chemistry with various biological activities, such as anti-microbial,^22–24^ anti-hypertensive,^25^ anti-diabetic,^26–28^ and anticancer agents.^29–32^ Hence, in this current work, our main aim is to design and synthesize novel ethyl 6-(chloromethyl)-1-methyl-2-oxo-4-phenyl-1,2,3,4-tetrahydropyrimidine-5-carboxylate derivatives as DNA intercalators and Topo-II inhibitors.

### 1.1. Rationale of the design

**Fig. 2:**
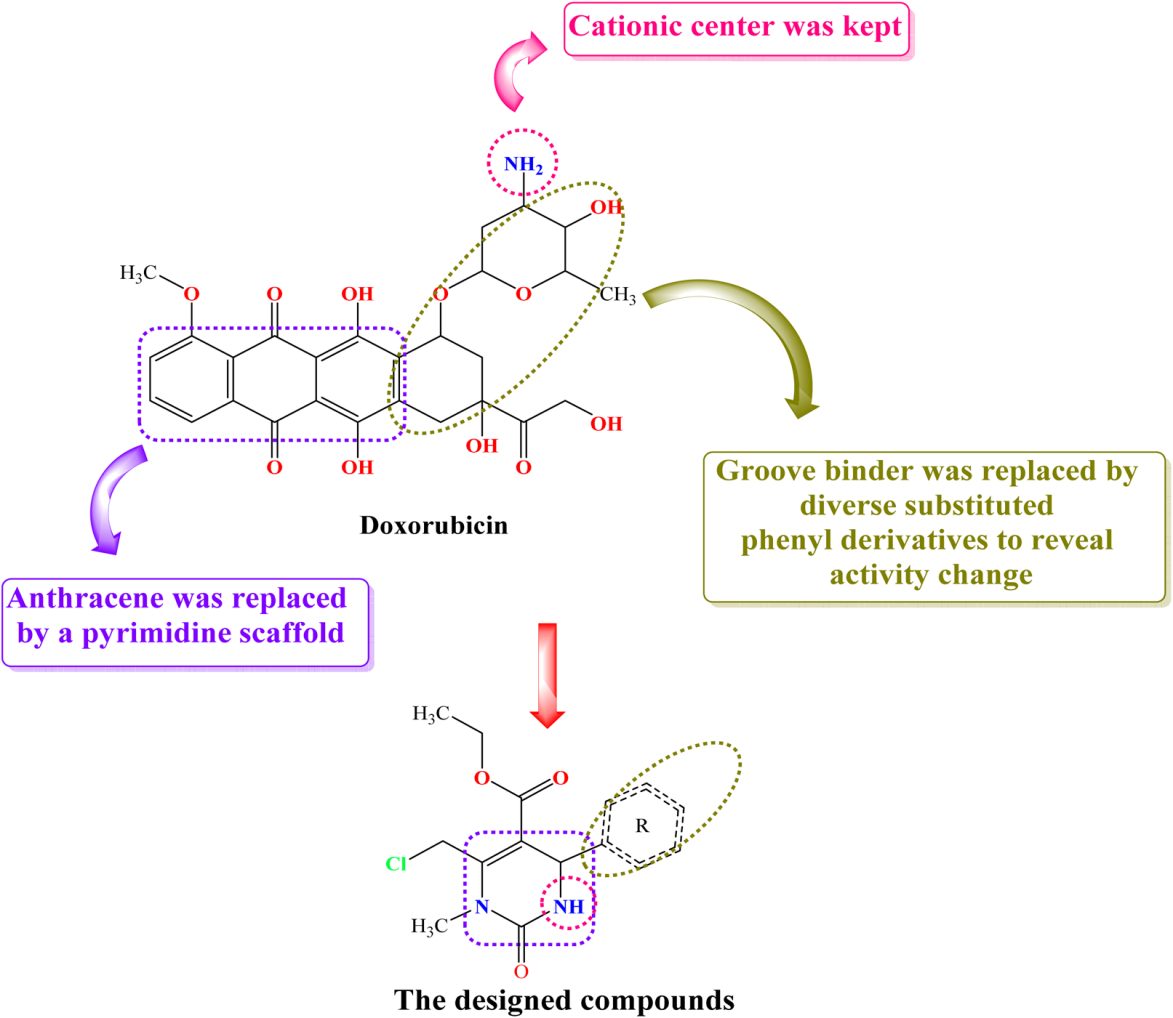
The design rationale of the investigated tetrahydropyrimidine derivatives illustrates the main pharmacophores of doxorubicin as a DNA intercalator and Topo-II inhibitor.

Herein, many DNA intercalators and Topo-II inhibitors, such as Dox, share key pharmacophores that are essential for their function. These pharmacophores include a planar polyaromatic chromophore, a groove binder (a component that fits into a specific area of the DNA molecule), and a positively charged region (cationic center).^10,33^ These structural features underpin the efficacy of DNA intercalators and Topo-II inhibitors by facilitating their interaction with designated regions of the DNA molecule. This interaction disrupts the enzyme's activity and consequently interferes with the maintenance of proper DNA topology, as the rational design is based on maintaining the main essential pharmacophores with the possibility of slight molecular modifications to pursue activity change. In this current work, we retained the main essential pharmacophores of Topo-II inhibitors and DNA intercalators with some molecular modifications for SAR studies. Moreover, we have considered the pharmacophoric nature of the parent compounds, not their exact chemical entities. Replacing the anthracene nucleus of Doxorubicin (Dox) with a tetrahydropyrimidine scaffold is a strategy rooted in medicinal chemistry and drug design, aiming to improve certain pharmacological properties, while retaining or modifying the therapeutic activity. For example, the quinone and aromatic rings in the anthracene nucleus are involved in redox cycling, generating reactive oxygen species (ROS) that damage cardiac tissue.^34^ Replacing this core with a less redox-active scaffold-like tetrahydropyrimidine could help minimize ROS generation and reduce cardiotoxic effects. So, the anthracene nucleus of Dox was replaced by a tetrahydropyrimidine scaffold (to be inserted in between the DNA base pairs, where both act as intercalators between the DNA nucleobases regardless of the difference in size between pyrimidine and anthracene scaffolds), the –NH_2_ group of Dox was kept in our designed compounds (to act as a cationic center that can be protonated at physiological pH to interact with the phosphate group of the DNA sugar moiety), and the groove binder of Dox was replaced by similarly acting diverse substituted phenyl motifs to be directed towards the minor groove of DNA (Fig. 2). It is worth noting that the 3,4,5-trimethoxy phenyl side chain as a groove binder can contribute to superior stability, lower RMSD, and better Δ*G* binding energy compared to Dox. Regarding stability, the 3,4,5-trimethoxyphenyl group can fit particularly well into the DNA minor groove, owing to the 1-steric compatibility (the narrow groove accommodates small, hydrophobic, and electron-rich aromatic rings), 2-hydrogen bonding potential (the methoxy groups can act as hydrogen bond acceptors), and the 3-hydrophobic interactions (groove interiors are relatively hydrophobic and the 3,4,5-trimethoxyphenyl non-polar surface matches this environment). Regarding RMSD, the 3,4,5-trimethoxyphenyl group side chain contributes to low RMSD because it locks into the groove *via* different interactions. Moreover, unlike Dox's intercalation, which can disturb the DNA geometry or flip out the bases, the groove binding of 3,4,5-trimethoxyphenyl is less invasive, so the ligand can stay more stable with lower RMSD values. The binding free energy (Δ*G*) may be more favorable in 3,4,5-trimethoxyphenyl-based groove binders since methoxy groups at positions 3, 4, and 5 can interact with DNA bases and sugar-phosphate backbone oxygens *via* hydrogen bonds. The phenyl ring can make tight van der Waals contacts with the groove wall, in addition to groove environments that favor non-polar interactions. The 3,4,5-trimethoxyphenyl group can achieve this type of interaction, affording favorable binding free energy (Δ*G*). In conclusion, our designed compounds retained the main pharmacophores of Dox required for DNA intercalation and Topo-II inhibition.

## 2. Results and discussion

### 2.1. *In silico* studies

#### 2.1.1. Molecular docking

**Scheme 1:**
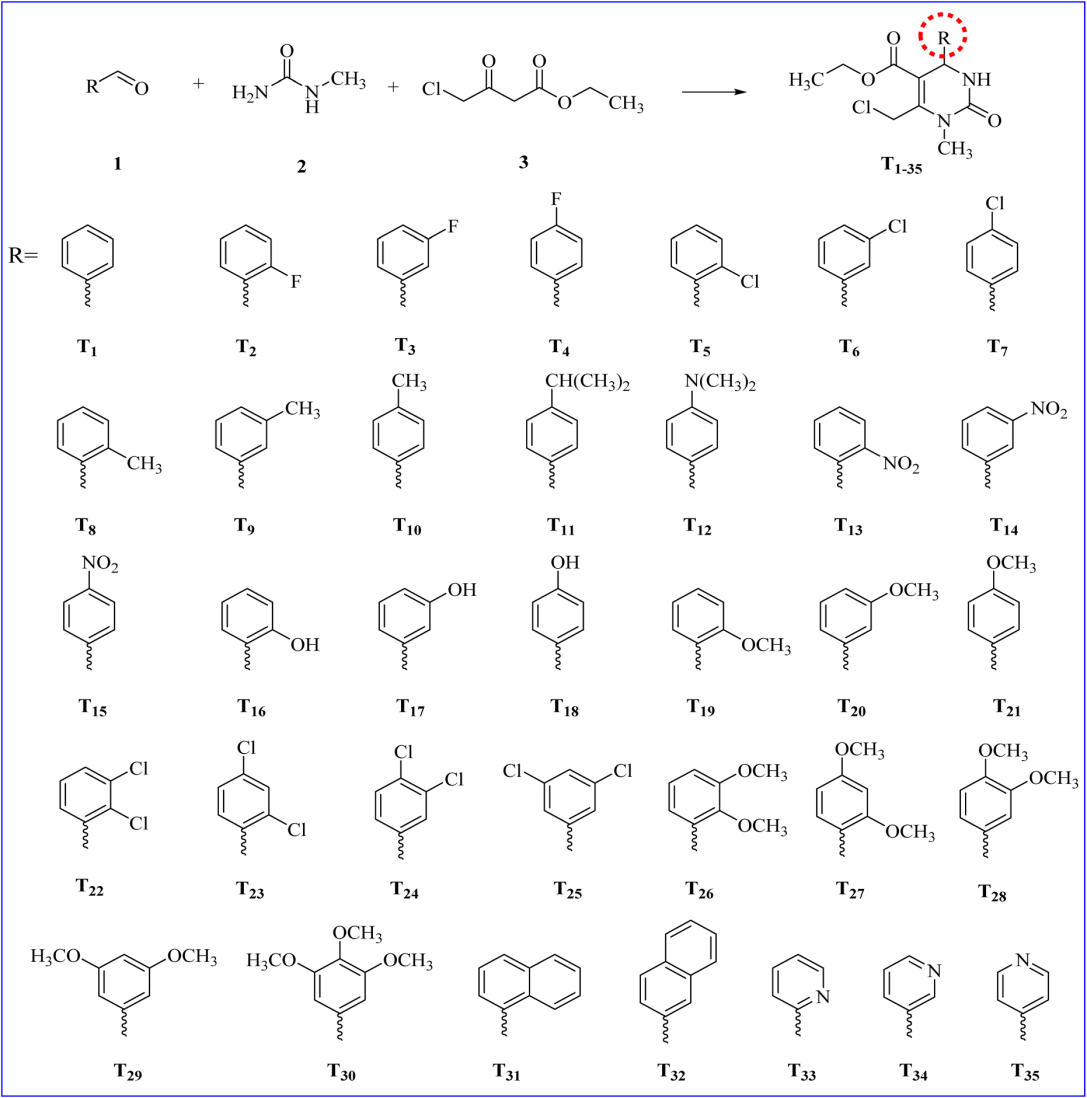
Cyclocondensation of different aromatic aldehydes 1 with ethyl 4-chloroacetoacetate 2 and *N*-methylurea 3 to obtain the target analogues (T_1–35_).

Based on the aforementioned rational design of DNA intercalators and Topo-II inhibitors, taking into consideration the main pharmacophoric features of Dox as a reference standard, our research team theoretically designed novel substituted tetrahydropyrimidine analogues (T_1–35_), as shown in Scheme 1.

These analogues were designed based on their novelty in synthesis and diversity. Afterward, all of the designed analogues (T_1–35_) were investigated for their inhibitory potential towards the hybrid DNA and Topo-II target receptor (PDB ID: 3QX3) using molecular docking. This was done to select the most promising analogue to be synthesized, and accordingly save effort, time, and cost in the drug discovery process. First, the active site residues were identified to be Met782, Gln778, Asp479, Lys456, Glu477, Gly478, Gly504, Leu502, Arg503, Ala817, DA12, DC11, DC14, and DG13. Moreover, both Dox and the co-crystallized inhibitor of the target receptor (EVP) were utilized as two reference standards.

Interestingly, the theoretically designed tetrahydropyrimidine analogue (T_30_) with a 3,4,5-trimethoxy phenyl side chain was found to be the superior candidate, achieving a binding score of −7.06 kcal mol^−1^ (RMSD = 1.56 Å). This binding score was comparable to both reference standards (Dox and EVP), which exhibited binding scores of −7.44 kcal mol^−1^ (RMSD = 1.58 Å) and −7.45 kcal mol^−1^ (RMSD = 1.57 Å), respectively. Additionally, the docking scores, RMSD, and binding interactions of the target novel substituted tetrahydropyrimidine analogues (T_1–35_) are summarized in the ESI data (Table S1†).

**Fig. 3:**
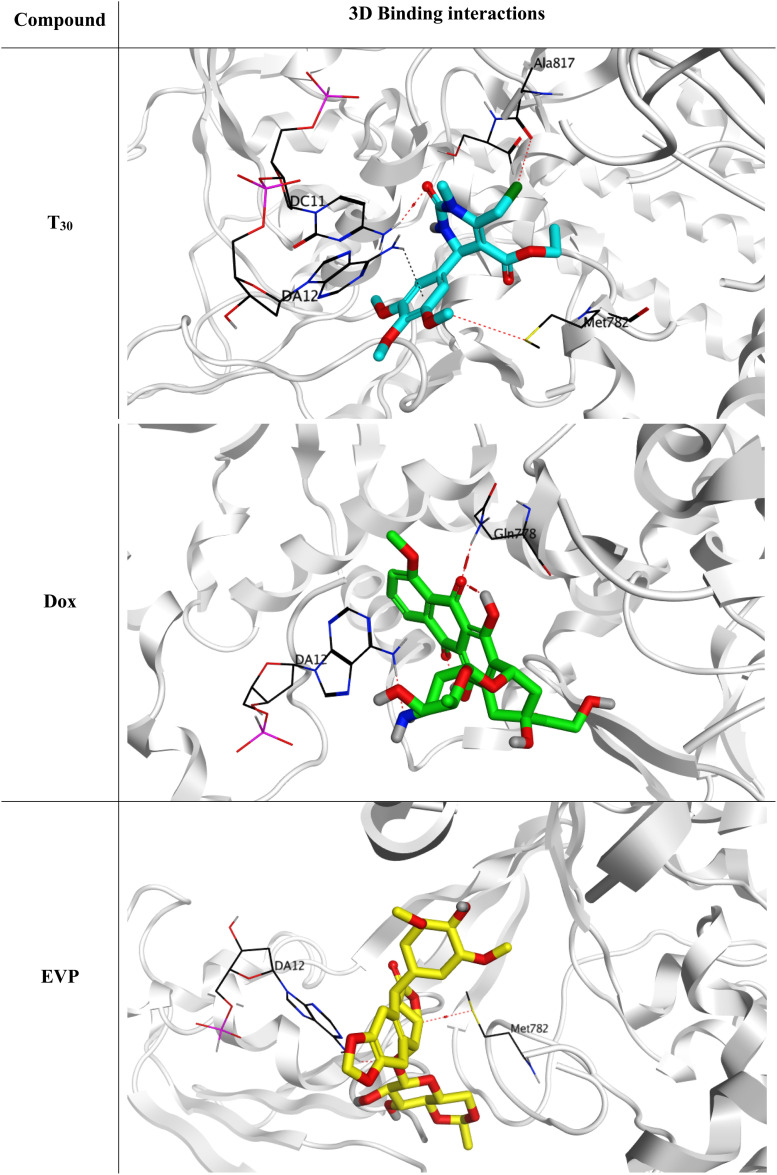
3D Binding interactions of the superior theoretically designed tetrahydropyrimidine analogue (T_30_), doxorubicin, and the docked co-crystallized EVP towards the hybrid DNA and Topo-II target receptor (PDB ID: 3QX3).

The binding mode of the designed analogue (T_30_) was highly similar to both reference standards (Dox and EVP), as shown in Fig. 3, illustrating the formation of one hydrogen bond with Met782 and one pi-hydrogen bond with DA12 through its trimethoxy phenyl side chain. Moreover, it formed two hydrogen bonds with DC11 and Ala817 through its tetrahydropyrimidine moiety extensions at C2 and C6. Accordingly, both hydrogen and pi-hydrogen bonds were clarified to be crucial types of interactions to stabilize analogue (T_30_) inside the binding pocket of DNA and the Topo-II target receptor. Moreover, Dox showed the formation of two hydrogen bonds with DA12 and Gln778. However, the docked co-crystallized EVP bound both DA12 and Met782 with two hydrogen bonds. These findings indicate the antagonistic potential of the designed analogue (T_30_) towards the hybrid DNA and Topo-II target receptor (PDB ID: 3QX3).

#### 2.1.2. Molecular dynamics simulation

Since the docking approach does not take into account the protein's flexibility, the docked candidates (T_30_, Dox, and EVP) were further subjected to molecular dynamics simulation for 500 ns to validate their stabilities within the active site of the hybrid DNA and Topo-II target receptor (PDB ID: 3QX3).

##### 2.1.2.1. RMSD analysis

**Fig. 4:**
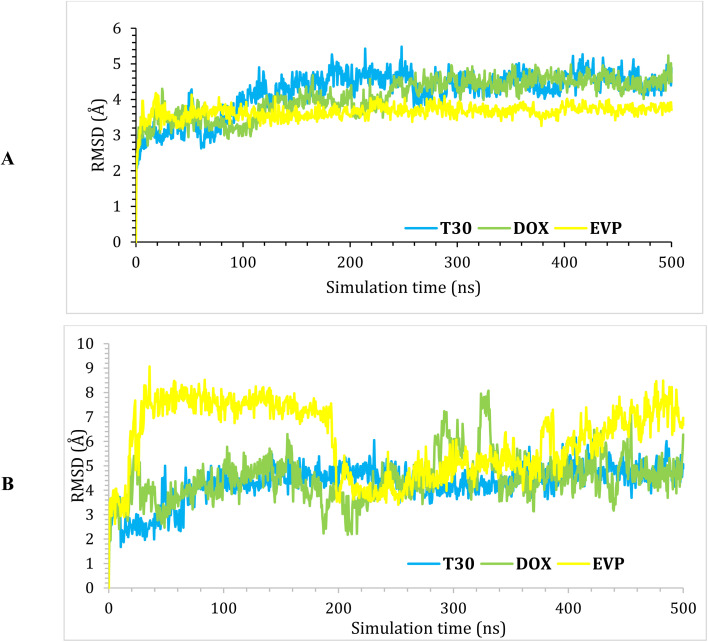
(A) Protein RMSD and (B) ligand RMSD for the complexes (T_30_, Dox, and EVP) of the hybrid DNA and Topo-II target receptor (PDB ID: 3QX3) binding domain as a function of simulation time (500 ns).

RMSD was performed to judge the system's stability. The RMSD describes the deviation degree of the complex protein with respect to its initial position in a quantitative way. The three examined complexes showed moderate stability with RMSD values <5.4 Å (Fig. 4A). Moreover, the ligand RMSD (L-RMSD) was studied to evaluate the exact behaviour of each ligand within the hybrid DNA and Topo-II target receptor (PDB ID: 3QX3) binding domain (Fig. 4B). Interestingly, compound T_30_ showed the most stable behaviour with an RMSD value of 6.3 Å as the maximum fluctuation level. However, Dox and EVP fluctuated to 8 and 9 Å, respectively, indicating their less stable behaviours compared to T_30_.

##### 2.1.2.2. Binding interactions histogram and heat map analysis

**Fig. 5:**
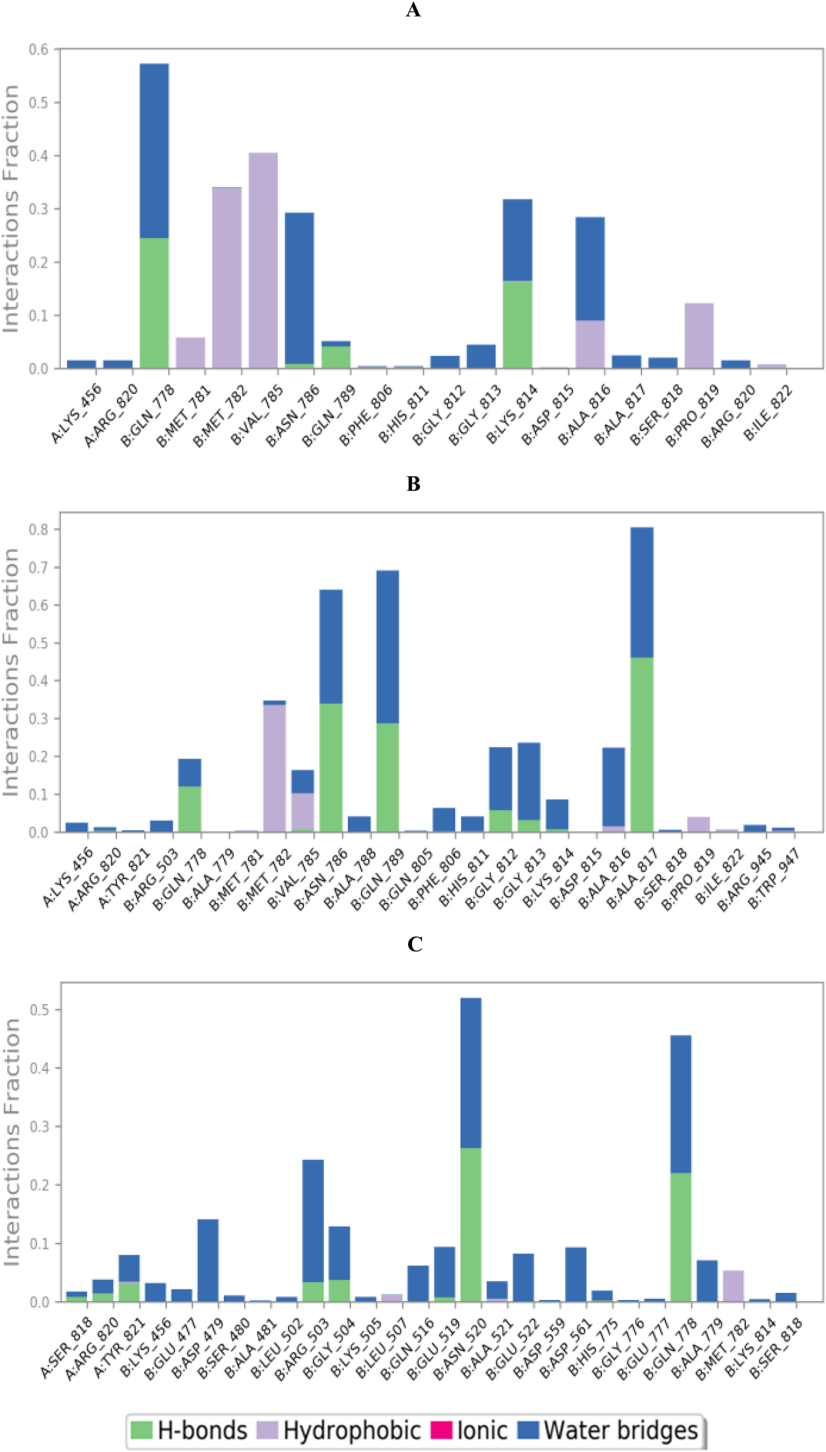
Histogram describing the binding interactions between the hybrid DNA and Topo-II target receptor (PDB ID: 3QX3) and its ligands: (A) T_30_, (B) Dox, and (C) EVP.

To completely describe the hybrid DNA and Topo-II target receptor (PDB ID: 3QX3)-ligand interactions, both histograms and heat maps were analyzed in detail (Fig. 5 and ESI Fig. S1†), respectively.

The histogram of the hybrid DNA and Topo-II target receptor in complex with compound T_30_ showed that Gln778 contributed the most to the interactions with about 58%. The types of interactions included hydrogen bonds and water bridges (Fig. 5A). However, Dox, Ala817, Gln789, and Asn786 contributed the most to the interactions at 80%, 70%, and 65%, respectively, with hydrogen bonds and water bridge interactions for the three amino acids (Fig. 5B). Moreover, the hybrid DNA and Topo-II receptor-EVP histogram showed that Asn520 and Gln778 were the superior amino acids with 52% and 45% contributions, respectively, through hydrogen bonds and water bridge interactions (Fig. 5C).

Briefly, it can be concluded that T_30_ shared the co-crystal (EVP) interactions with Gln778 amino acid, which may be considered the most crucial residue to produce the antagonistic activity towards the hybrid DNA and Topo-II receptor.

Furthermore, the exact time of the binding interactions for the hybrid DNA and Topo-II target receptor residues with T_30_, Dox, and EVP candidates were represented by heat map output (ESI Fig. S1†).

The heat map of the hybrid DNA and Topo-II target receptor-T_30_ complex showed that the interactions of Gln778 were more prominent after 100 ns of the simulation time, and were preserved until the end of the simulation (Fig. S1,† A). Conversely, more intense behaviours were observed for Ala817, Gln789, and Asn786 interactions with Dox (Fig. S1B†). The interactions of Ala817 were more intense in the first half of the simulation time. In contrast, the interactions of Gln789 and Asn786 were more intense in the second half of the simulation time. Additionally, the hybrid DNA and Topo-II target receptor-EVP complex heat map showed that Asn520 and Gln778 continually contributed to the binding interactions throughout most of the simulation time (Fig. S1C†).

#### 2.1.3. MM-GBSA calculations

MM-GBSA calculations using the thermal_mmgbsa.py python script of Schrodinger^35^ were carried out for all complexes (T_30_, Dox, and EVP)-3QX3, and are summarized in Table 1. Notably, the target candidate (T_30_) achieved superior Δ*G* binding energy (−33.86 kcal mol^−1^) compared with Dox and EVP (−33.80 and −27.94 kcal mol^−1^, respectively), indicating the more favorable binding affinity of the tetrahydropyrimidine scaffold of T_30_ than that of the anthracene core of Dox.

**Table 1:** MM-GBSA energies for the complexes (T_30_, Dox, and EVP) of the hybrid DNA and Topo-II target receptor^a^

Complex	Δ*G* binding	Coulomb	Covalent	H-bond	Lipo	Bind packing	Solv_GB	VdW
T_30_	−33.86	−963.07	6.41	−0.43	−10.79	−0.39	1024.80	−90.38
Dox	−33.80	−1046.22	5.18	−0.50	−13.04	−0.51	1133.57	−112.27
EVP	−27.94	−930.40	6.02	−0.26	−10.52	−0.06	998.07	−90.79

a Coulomb: Coulomb energy; covalent: covalent binding energy; H-bond: hydrogen-bonding energy; lipo: lipophilic energy; Solv_GB: generalized born electrostatic solvation energy; VdW: van der Waals energy; and St. Dev.: standard deviation.

### 2.2. Chemistry

**Scheme 2:**
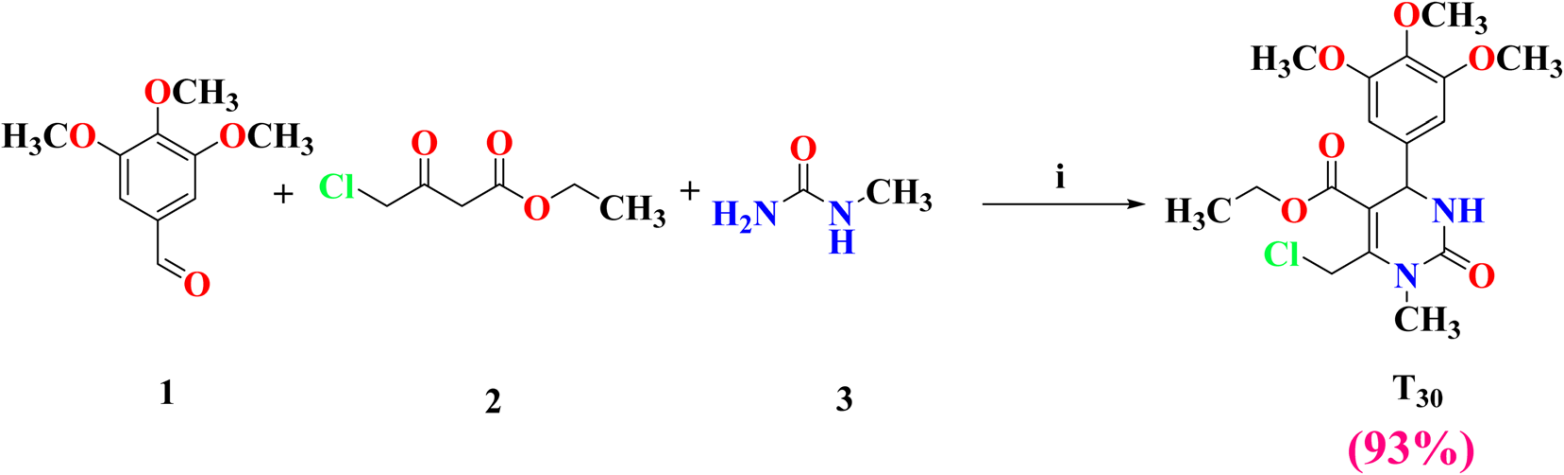
Synthesis of the THPM derivative T_30_. Reagents and conditions: (i) citric acid, TEOF, absolute ethanol, and reflux 10 h.

As presented in Scheme 2, the most promising candidate (T_30_) was conveniently synthesized through Biginelli multicomponent reaction (MCR).^36,37^ It involves the cyclocondensation of 3,4,5-trimethoxybenzaldehyde 1 with ethyl 4-chloroacetoacetate 2 and *N*-methylurea 3 in reflux with absolute ethanol, in addition to citric acid and triethylorthoformate (TEOF) to remove the water molecule and the formation of the tetrahydropyrimidine (THPM) analogue T_30_ with high yield (93%).

Elemental analysis and spectroscopic data (^1^H NMR, ^13^C NMR, DEPTQ, HMQC, mass spectroscopy, and HPLC) of the newly synthesized compound were in agreement with the expected chemical structure, as reported in the experimental section. Generally, the ^1^H NMR spectral data of the target compound showed a pair of doublet signals at *δ* 5.08 and 8.06 ppm, representing 4-H and 3-NH, respectively, at the THPM ring, and the disappearance of the singlet signals of proton of the starting aldehyde and the two protons of the active methylene (–CH_2_) of ethyl 4-chloroacetoacetate 2 at around *δ* 9.82 and 3.41 ppm, respectively. ^13^C NMR spectra showed the presence of 4-C at the THPM ring at *δ* 52.59 ppm, the disappearance of the carbonyl group of the starting aldehyde, and one of the two carbonyl groups of ethyl 4-chloroacetoacetate 2 in the expected range of *δ* 190–210 ppm. The conducted HRMS further assured the chemical structure of compound T_30_ (cald./found 399.13174/399.13194 *m/z*).

The ^13^C NMR-DEPTQ approach was used to discriminate between carbons that carried an odd number of protons (CH and CH_3_) and those bearing an even number of protons or without protons (CH_2_ and C_Q_) by revealing signals upfield (positive) and downfield (negative), respectively, to the baseline. Compound T_30_ showed two aliphatic peaks downfield to the baseline at 38.50 and 60.91 ppm, respectively, for the CH_2_Cl and CH_2_O groups, in addition to all quaternary carbons. In addition, it showed peaks above the baselines related to all CH_3_ groups (three OCH_3_, NCH_3_, and OCH_2_CH_3_) and all CH groups (two benzene CH at positions 2/6 and one THPM CH at position 4).

**Fig. 6:**
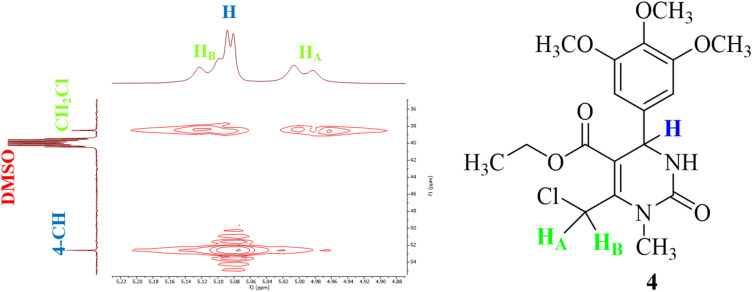
^1^H–^13^C HMQC NMR spectrum of compound T_30_ in the regions (^1^H) 4.87–5.23 ppm and (^13^C) 35–55 ppm.

Furthermore, the two-dimensional ^1^H–^13^C HMQC (Heteronuclear Multiple Quantum Coherence) technique was used to correct and complete the ^13^C NMR peak assignments. From the ^1^H–^13^C HMQC spectrum shown in Fig. 6, two separate cross-peaks can easily be seen by correlation of proton H_A_ at 4.99 ppm with the carbon signal at 38.49 ppm, and that of proton H_B_ at 5.11 ppm with the carbon signal at the same chemical shift (38.49 ppm). Based on this, the signal at 38.49 ppm could easily be assigned to the carbon atom attached to both protons (H_A_ and H_B_) of compound T_30_. The previous conclusion was supported by the same coupling constant of both doublet signals at 4.99 and 5.11 ppm (*J* = 10.0 Hz). Although there is a third doublet signal for a proton in that region (5.08 ppm), it was possible to conclude that it couples with the doublet signal of the NH group (8.06 ppm) because of the same coupling constant value (*J* = 5.0 Hz). Thus, we can conclude that it is the proton belonging to the carbon number four at the THPM ring.

The mass spectra of the synthesized compound showed a peak corresponding to the molecular ion and its isotope. According to HPLC analysis, compound T_30_ has a purity level greater than 98.9% (see the purity value listed for the lead compound in the experimental section and Fig. S7†).

### 2.3. Biological evaluation

#### 2.3.1. Evaluation of *in vitro* antiproliferative activity by NCI

In order to determine the inhibitory effect and range of compound T_30_, we submitted a request to the National Cancer Institute (NCI) to evaluate its efficacy against a selection of nine different types of cancer cells. NCI employs a total protein assay using sulforhodamine B (SRB), a widely recognized method noted for its high sensitivity, accuracy, and reduced vulnerability to interferences generated by the tested chemical. This is due to its independence from cellular metabolic activities.^3,38,39^ Compound T_30_ was tested against various cancers, including blood, lung, colon, CNS, skin, ovary, renal, prostate, and breast, using a single dose (10 μM) and five doses (GI_50_) (0.01, 0.1, 1, 10, and 100 μM).

Compound T_30_ exhibited significant antiproliferative activity, with mean GI% = 122% and mean GI_50_ = 4.10 μM. It had the highest level of effectiveness against all 59 cell lines in terms of inhibiting cancer growth. Compound T_30_ demonstrated deadly effects in 39 instances of cancer cells, with growth inhibition percentages ranging from 101% to 196% (Fig. S8†). In addition, it exhibited remarkable activity with GI_50_ values ranging from 1.43 to 13.5 μM (6 of them being less than 2.00 μM) and LC_50_ values ranging from 7.16 to greater than 100.0 μM (Fig. S9†).

#### 2.3.2. *In vitro* assessment of the effectiveness against blood malignancies

**Fig. 7:**
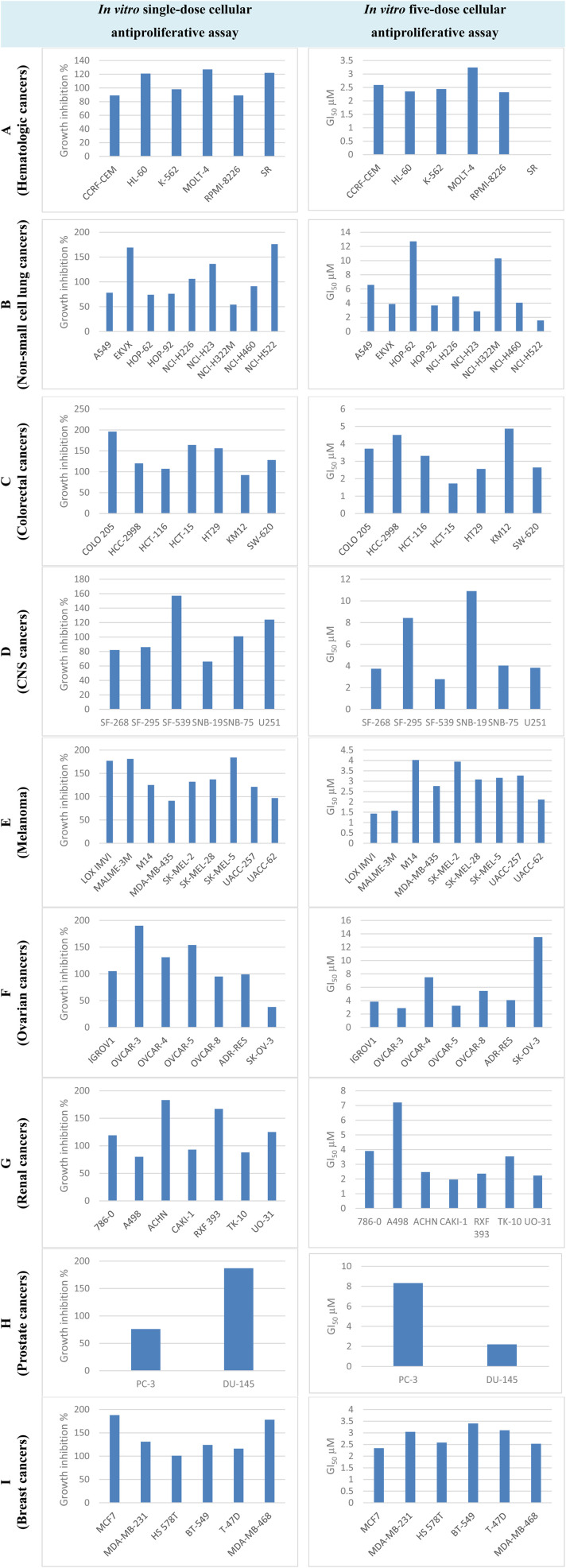
GI% and GI_50_ of cell lines representing diverse cancer subtypes by compound T_30_.

Haematologic malignancies, although classified as a single cancer group, exhibit diversity and can be classified into three subtypes based on their origins: leukaemia, lymphoma, and multiple myeloma. A recent study has uncovered that haematologic cancer patients experience a decline in their quality of life due to both the blood cancer itself and the adverse effects of current medications. Consequently, there is a need for improved treatments to meet these unresolved challenges.^40^ Considering this, we investigated the effectiveness of the synthesized compound T_30_ on several cell lines associated with leukaemia (HL60 and K562), lymphoma (CCRF-CEM, MOLT-4, and SR), and multiple myeloma (RPMI8226) using one-dose and five-dose treatments. The findings are presented in Fig. 7A. Compound T_30_ exhibited deadly effects on HL-60, MOLT-4, and SR, with growth inhibition percentages (GI%) of 121, 127, and 122, respectively. All blood cancer cell lines displayed a GI_50_ range of 2.24–2.59 μM. The RPMI-8226 cell line exhibited the highest sensitivity to T_30_ among the investigated cell lines.

#### 2.3.3. *In vitro* assessment of the effectiveness against non-small cell lung malignancies

Lung cancer is the leading cause of mortality worldwide among all types of cancer. It is categorized into two main types: small-cell lung cancer (SCLC) and non-small-cell lung cancer (NSCLC). SCLC, which constitutes more than 85% of lung cancers, is less responsive to chemotherapy compared to the former. NSCLCs are categorized into adenocarcinoma, squamous cell carcinoma, or large-cell carcinoma. The synthesized hybrid compounds were subsequently evaluated against a panel of nine non-small cell lung cancers (NSCLCs), encompassing lung adenocarcinoma cells (A549, EKVX, HOP62, HOP92, H23, and H522), metastatic lung adenocarcinoma (H322 cell line), large cell lung carcinoma (H460 cell line), and squamous carcinoma (H226 cell line). The findings are succinctly presented in Fig. 7B. Compound T_30_ exhibited a wide range of effectiveness, with a growth inhibition percentage (GI%) ranging from 54 to 176 and a GI_50_ (concentration required for 50% growth inhibition) ranging from 1.55 to 12.7 μM. The lung cancer cell that exhibits the highest sensitivity is H522, which is classified as adenocarcinoma and has a GI% of 176 (indicating deadly activity) and a GI_50_ of 1.55 μM. Conversely, the two cells that are least affected are HOP-62 and H322M. The NCI-H522 cell line was most sensitive to T_30_ among the investigated cell lines.

#### 2.3.4. *In vitro* assessing the effectiveness against colorectal malignancies

Colon cancer is quite prevalent, with adenocarcinomas comprising around 95% of all instances. Although originating from the same histology source, each colon cancer cell line has individual and distinguishable molecular and genetic alterations.^41^ Consequently, we tested our drug on a group of seven colon cancer cell lines, and the outcomes are displayed in Fig. 7C. Compound T_30_ showed high effectiveness and deadly activity against all tested colon cancer cell lines, with GI% values ranging from 107 to 196 (except for KM12 with a GI% of 92). Additionally, the compound exhibited a single-digit GI_50_ value of less than 5 μM, ranging from 1.72 to 4.87. The HCT-15 cell line was the most sensitive to T_30_ among the investigated cell lines.

#### 2.3.5. *In vitro* assessment of the effectiveness against CNS malignancies

CNS tumours are categorized into two groups: gliomas and non-gliomas. Among them, glioblastoma multiforme (GBM) brain malignancies are the most prevalent and exhibit high resistance to treatment. The resistance is associated with the diversity of the cancer cells, the repeated occurrence of resistant forms, and the strict criteria that the chemotherapy drug must fulfill to effectively penetrate the blood–brain barrier.^42^ The effectiveness and range of activity of the synthesized compound were evaluated against a group of six GBM cell lines. The results of this evaluation can be found in Fig. 7D. Compound T_30_ demonstrated significant growth inhibition (GI) against the majority of the examined glioblastoma multiforme (GBM) cell lines. The GI percentage ranged from 66% to 157%, while the GI_50_ values ranged from 2.78 to 10.9 μM. The CNS cancer cell line that is most vulnerable to compound T_30_ is SF-539, with a GI% of 157 and a GI_50_ of 2.78 μM. On the other hand, the least sensitive cell line is SNB-19, with a GI% of 66 and a GI_50_ of 10.9 μM. The SF-539 cell line was most sensitive to T_30_ among the investigated cell lines.

#### 2.3.6. *In vitro* assessment of the effectiveness against melanoma malignancies

Melanoma is the predominant form of skin cancer and is notoriously challenging to treat.^43^ The inefficiency of existing chemotherapeutics is caused by the presence of intratumoral and intertumoral heterogeneity in melanomas.^44^ To further advance the development of new treatments for melanoma, the synthesized molecule's effectiveness and range of activity were assessed by testing it on a group of nine melanoma cell lines. The outcomes of this experiment are presented in Fig. 7E. Compound T_30_ exhibited significant growth inhibition percentages (GI%) ranging from 97 to 184, as well as GI_50_ values ranging from 1.43 to 4.02 μM, against nearly all of the nine melanoma cell lines used in the study. Notably, it showed particularly strong activity against the LOXIMVI cell line, with a GI% of 177 and a GI_50_ of 1.43 μM, as well as the MALME-3M cell line, with a GI% of 181 and a GI_50_ of 1.57 μM. The LOX IMVI cell line was the most sensitive one to T_30_ among the investigated cell lines.

#### 2.3.7. *In vitro* assessing the effectiveness against ovarian malignancies

Ovarian cancers are known for their high mortality rates, with just 30% of patients surviving for five years and 70% experiencing a recurrence. The average duration of progression-free survival is approximately one to one and a half years.^45^ Epithelial ovarian malignancies constitute around 95% of all cases of ovarian cancer,^46^ despite the existence of several other kinds. Regrettably, platinum-based chemotherapeutics, known for their harshness and resistance, continue to be the standard post-operative treatment.^47^ Several targeted medications are available for the treatment of ovarian cancer, such as antiangiogenic and PARP inhibitors.^48^ Consequently, there is a pressing necessity to uncover novel therapies for ovarian cancer. We conducted an experiment where we evaluated the effectiveness of our synthesized compound on a group of seven ovarian cancer cells. The results of this experiment can be observed in Fig. 7F. Compound T_30_ inhibited the growth of six out of the seven ovarian cancer cell lines tested, reducing their growth by 95 to 190 percent and with a GI_50_ range of 2.88 to 13.5 μM. Notably, it was particularly effective against the OVCAR-3 cell line, with a growth inhibition of 190 percent and a GI_50_ of 2.88 μM. The SK-OV-3 cell line is relatively resistant to ovarian cancer, as indicated by its low susceptibility with a growth inhibition percentage (GI%) of 38. Furthermore, the GI_50_ value for this cell line is greater than 10 μM, suggesting that a higher concentration of a particular substance is required to inhibit its growth. The OVCAR-3 cell line was most sensitive to T_30_ among the investigated cell lines.

#### 2.3.8. *In vitro* assessment of the effectiveness against renal malignancies

Renal cell carcinomas account for the majority (92%) of kidney cancer cases.^49^ The prevalence of renal cell carcinoma is continuously rising. Moreover, the diverse nature of renal cell carcinomas limits the effectiveness of therapy and results in the development of resistant tumours.^50^ Consequently, there is a need for novel compounds to offer alternate options to the existing anticancer drugs for the treatment of kidney cancer. The hybrid compounds that were developed were evaluated against a panel of eight renal cell carcinomas to assess their effectiveness and range. The results of this testing are shown in Fig. 7G. Compound T_30_ demonstrated fatal action ranging from 119% to 183% against four renal cancer cell lines, and exhibited excellent growth inhibition percentages (GI%) ranging from 80% to 93% against three other renal cancer cell lines. Furthermore, it exhibited encouraging single-digit GI_50_ readings ranging from 1.78 to 7.20 μM. The CAKI-1 cell line was most sensitive to T_30_ among the investigated cell lines.

#### 2.3.9. *In vitro* assessment of the effectiveness against prostate malignancies

Small cell prostate carcinomas (SCPC) are the most lethal and refractory to antihormonal therapy targeting androgen deprivation. Usually, their reaction to chemotherapy is short-lived, and the typical survival duration is as little as one year.^51^Fig. 7H demonstrates the effectiveness of the synthesized medication against two highly aggressive cell lines, PC3 and DU145. The findings demonstrated that compound T_30_ significantly suppressed the proliferation of DU-145 cells to a greater extent than PC-3 cells, as indicated by the GI% and GI_50_ values, which were more than four times higher. The DU-145 cell line was the most sensitive one to T_30_ among the investigated cell lines.

#### 2.3.10. *In vitro* assessment of the effectiveness against breast malignancies

Among adult females under the age of 60, breast cancer is responsible for a considerable number of cancer-related deaths.^52^ Certain types of breast cancers exhibit positive expression of estrogen, progesterone, or Her2, whereas others do not, known as triple-negative breast cancers (TNBCs). The distinctive characteristics of TNBC render targeted and hormonal therapies ineffectual.^53^ Moreover, TNBCs often have a high level of aggressiveness and invasiveness. Consequently, our series underwent testing using an NCI panel of breast cancer cells, which consisted of two estrogen receptor-positive breast cancer cell lines (MCF7 and T47D) and four TNBC cell lines (MDA-MB-231, HS578T, BT549, and MDAMB468). The findings are presented in Fig. 7I. Compound T_30_ demonstrated fatal action against all kidney cancer cell lines, increasing their activity by 101–188%. Additionally, it showed outstanding GI_50_ findings ranging from 2.34–3.40 μM, with the MCF7 cell line being particularly responsive (GI% = 188 and GI_50_ = 2.34 μM). The MCF-7 cell line was the most sensitive one to T_30_ among the investigated cell lines.

### 2.4. Spectroscopic approaches

#### 2.4.1. DNA-binding study

The objective of this work was to obtain a comprehensive overview of how compound T_30_ binds to ctDNA to confirm its potential as an anticancer agent, as indicated by the results of *in vitro* cytotoxic tests.

#### 2.4.2. Study of the binding manner

##### 2.4.2.1. UV spectrophotometric study

**Fig. 8:**
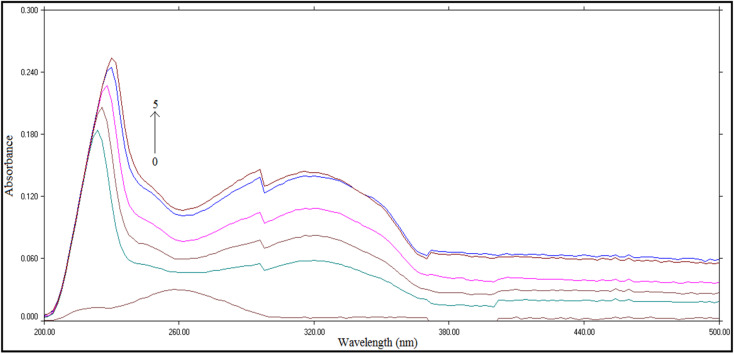
UV spectrophotometric spectra of ctDNA (97.0 μM) with increasing concentrations of compound T_30_ from 0 to 5 (0, 5.0, 10.0, 20.0, 30.0 and 40.0) μM.

This study relies on the addition of increasing concentrations of compound T_30_ to ctDNA and observing the alterations in the intensity and position of the DNA's distinctive peak at 260 nm, which is associated with the π–π* transition of the base pairs of DNA.^54^ The study revealed that the intensity of the ctDNA peak increased as the concentration of compound T_30_ increased, as depicted in Fig. 8. Furthermore, the peak position exhibited a red shift towards a shorter wavelength. Consequently, based on the shift rule of the DNA distinctive absorption peak, the binding process is anticipated to occur through intercalation mode rather than groove.^55^

##### 2.4.2.2. Viscosity studies

**Fig. 9:**
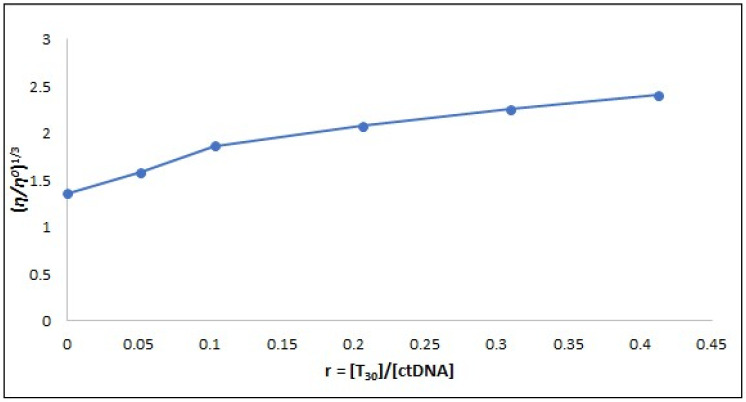
Effect of different concentrations of compound T_30_ (0–50.0 μM) on the ctDNA viscosity (97.0 μM) in Tris–HCl buffer at 298 K.

Viscosity studies are crucial for investigating alterations in the DNA length and how it binds.^56^ The traditional intercalative binding process significantly affects the viscosity of the DNA solution due to its requirement for ample space between adjacent base pairs to elongate the double helix and accommodate micro-molecules.^57^ Conversely, the impact of the electrostatic and groove binding processes on the DNA viscosity is minimal or nonexistent, as supported by many studies.^58,59^ The data presented in Fig. 9 demonstrates that the relative specific viscosity of ctDNA (*η/η*^0^)^1/3^ varies on subsequent addition of compound T_30_, providing evidence for the intercalation binding mode of compound T_30_ with ctDNA. This finding is in line with the results obtained from the spectrophotometric investigation.

##### 2.4.2.3. Spectrofluorimetric competitive binding interactions

**Fig. 10:**
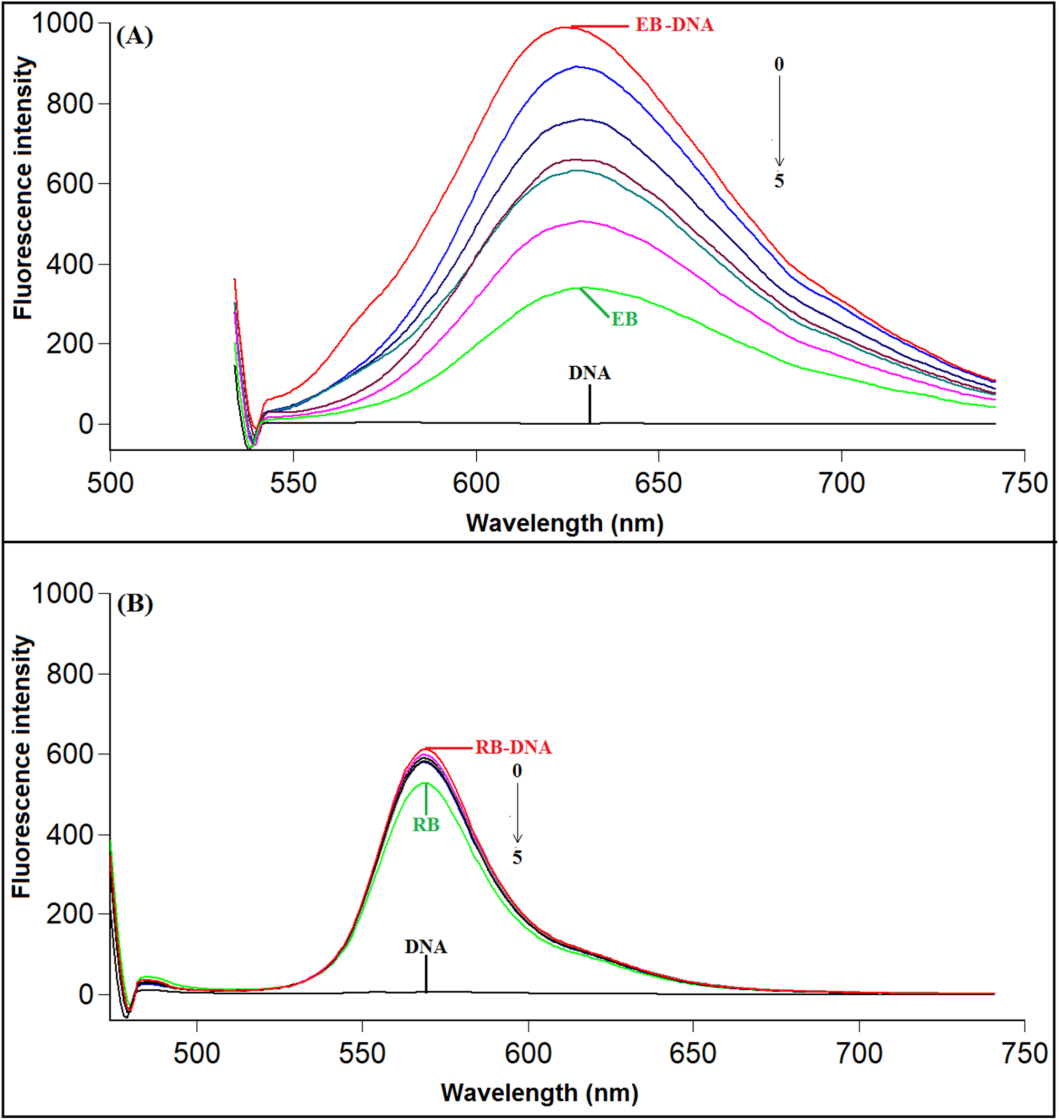
(A) Fluorescence emission spectra of the EB-ctDNA complex in the presence and absence of compound T_30_ at 298 K. C (ctDNA): 97.0 μM; C (EB): 7.0 μM; C (compound T_30_): 0, 5.0, 10.0, 20.0, 30.0, 40.0 μM, (*λ*_ex_/*λ*_em_ = 525/603 nm). (B) Fluorescence emission spectra of the RB-ctDNA complex in the presence and absence of compound T_30_ at 298 K. C(ctDNA): 97.0 μM; C(RB): 1.27 μM; C (compound T_30_) (0 → 5): 0, 5.0, 10.0, 20.0, 30.0, 40.0 μM, (*λ*_ex_/*λ*_em_ = 465/576 nm).

Competitive binding studies were carried out utilizing Rhodamine B (RB) and Ethidium bromide (EB) fluorescent probes to investigate the binding of compound T_30_ to ctDNA. According to reports, RB binds to the DNA's minor groove, particularly in areas rich in AT base pairs. In contrast, EB binds to DNA by intercalation.^60,61^ The fluorescence intensities of EB and RB increase when they attach to DNA.^62^ The analysis revealed that the fluorescence intensity of the EB-DNA complex decreased as the concentration of compound T_30_ increased. However, there was no impact on the fluorescence intensity of the RB-DNA complex, which further supports the intercalation binding process (Fig. 10).

##### 2.4.2.4. Ionic strength evaluation

**Fig. 11:**
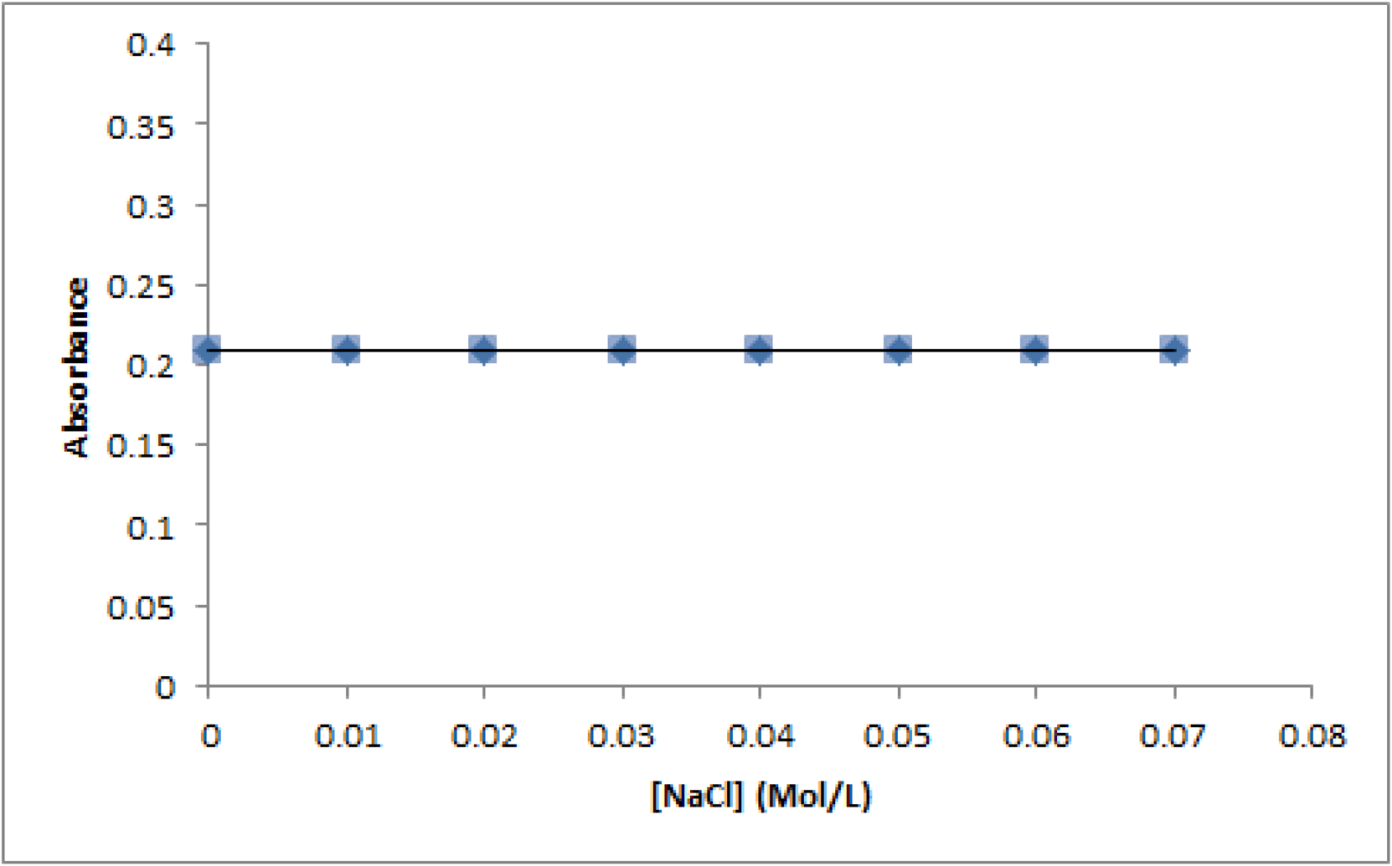
Influence of the NaCl ionic strength on the absorbance of the ctDNA-compound T_30_ complex. The conc. of compound T_30_ and ctDNA were 2.0 μM and 97.0 μM, respectively. The conc. of NaCl was 0–0.07 M.

The degree of electrostatic contact is substantially influenced by the ionic strength of the reaction media.^63^ Thus, in order to assess the potential for electrostatic interaction between ctDNA and compound T_30_, the impact of different NaCl concentrations on their binding relationship was examined. The data presented in Fig. 11 demonstrate that the absorbance of the complex with ctDNA remains constant as the concentration of NaCl increases from 0 to 0.07 M. This suggests the absence of any electrostatic interaction. Briefly, the previously revealed experimental data demonstrate an intercalation binding interaction between compound T_30_ and ct-DNA.

#### 2.4.3. Estimation of binding affinity between compound T_30_ and ctDNA

Establishing the binding affinity of a drug to a biomacromolecule is crucial because the drug's effectiveness is directly linked to its binding affinity. The dissociation constant (*K*_d_) or binding constant (*K*_b_) can be utilized to approximate the strength of binding. The Benesi-Hildebrand eqn (1) can be used to calculate the binding constant (*K*_b_) of the 1 : 1 complex formed between compound T_30_ and ctDNA:^64^1
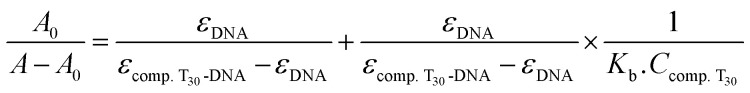
where: *A* and *A*_0_ are the ctDNA absorbance with or without compound T_30_, respectively. *ε*_DNA_ and *ε*_Comp. − DNA_ signify the molar extinction coefficients of ctDNA and the ctDNA-compound T_30_ complex, respectively. *C*_compound_T_30_ is the concentration of compound T_30_.

**Fig. 12:**
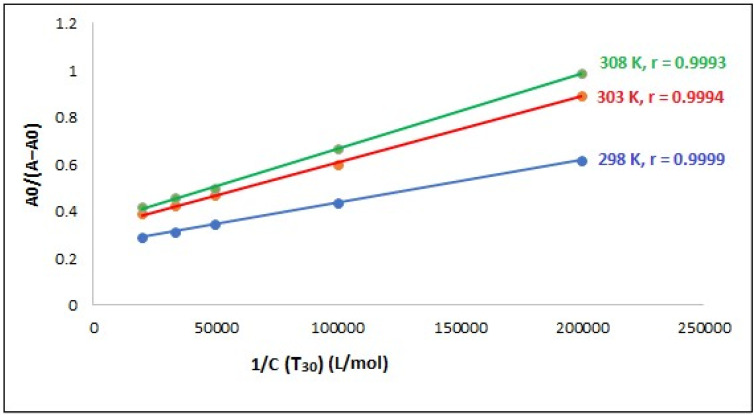
A plot of *A*_0_/(*A* − *A*_0_) *versus* 1/*C*_compound_T_30_ at different temperature settings (*C*_DNA_ = 97.0 μM), where *r* is the correlation coefficient.

A linear relationship was observed when plotting 1/*C*_compound 4_*versus A*_0_/(*A* − *A*_0_) at various temperatures (298, 303, 308 K), as shown in Fig. 12. This outcome indicates that the resultant complex exhibits a stoichiometry of 1 : 1. Table 2 displays the various *K*_b_ values acquired for the resultant complex utilizing eqn (1). The *K*_b_ values were in the order of 10^6^ M^−1^, indicating that compound T_30_ has a significant affinity for binding to ctDNA. In addition, the values seem to be comparable to those of the intercalators, with a *K*_b_ of 1.4 × 10^6^ M^−1^ (ref. 65) and higher than groove binders,^61^ demonstrating the involvement of the intercalation binding process in the binding of ctDNA-compound T_30_. Furthermore, when the temperature increases, the binding affinity between ctDNA and compound T_30_ increases, suggesting that the interaction between compound T_30_ and ctDNA is an endothermic process.^54^

**Table 2:** Assessment of the binding constants (*K*_b_) of the ctDNA-compound T_30_ complex and thermodynamic parameters at the studied temperatures

*T* (K)	*K*_b_ (M^−1^)	Δ*S*° (J mol^−1^ K^−1^)	Δ*H*° (kJ mol^−1^)	Δ*G*° a (kJ mol^−1^)	Δ*G*° b (kJ mol^−1^)
298	1.45 × 10^6^	182.97	19.40	−35.157	−35.125
303	1.60 × 10^6^			−35.998	−36.04
308	2.10 × 10^6^			−37.284	−36.95

a Δ*G*° = *RT* ln *K*_*b*_. in (kJ mol^−1^)

b Δ*G*° = Δ*H*° – *T*Δ*S*° in (kJ mol^−1^)

#### 2.4.4. Estimation of thermodynamic factors and key binding forces

The contact between small molecules and biomacromolecules involves four non-covalent binding forces: hydrogen bonding, hydrophobic, electrostatic forces, and van der Waals. Additionally, the nature of binding forces can be deduced by examining the sign and amount of entropy (Δ*S*°) and enthalpy (Δ*H*°) changes. When the values of Δ*S*° and Δ*H*° are positive, hydrophobic interaction becomes the prevailing force. However, when Δ*S*° is positive and Δ*H*° is nearly zero, electrostatic interaction becomes the primary force. Hydrogen bonding and/or van der Waals interactions are the main interaction attractions when both Δ*S*° and Δ*H*° are negative.^66,67^ The van't Hoff eqn (2) and (3) were utilized to compute the thermodynamic parameters for the binding of compound T_30_ with ctDNA, which comprised the Gibbs free energy change (Δ*G*°), Δ*H*°, and Δ*S*°:^67^2
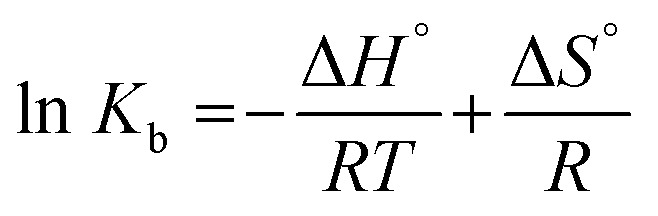

3 Δ*G*° = Δ*H*° − *T*Δ*S*°

where *R* represents a gas constant.

**Fig. 13:**
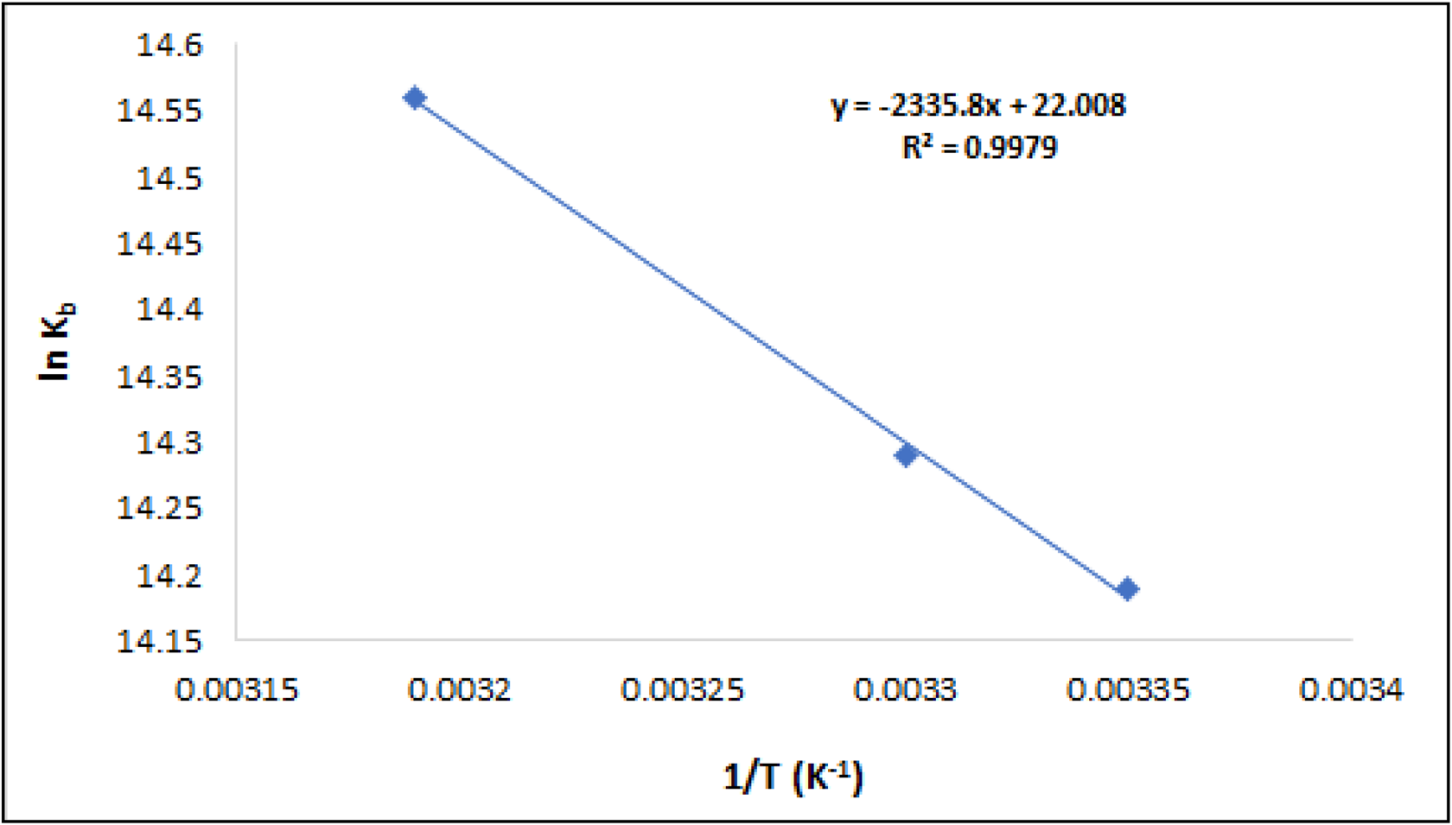
Vanʼt Hoff graph for the ctDNA-compound T_30_ complex.

The graph depicted in Fig. 13 illustrates the relationship between ln *K*_b_ and 1/*T*, according to Van't Hoff's equation. The plot's slope and intercept were utilized to estimate Δ*H*° and Δ*S*°, correspondingly, which are shown in Table 2. The presence of hydrophobic contacts typically leads to positive values of Δ*H*° and Δ*S*°. Since the result of Δ*H*° was greater than zero, it provided further confirmation that the binding process was endothermic. Furthermore, it was found that Δ*G*° is negative, indicating a spontaneous binding association. Therefore, the process of compound T_30_ binding with ctDNA is both endothermic and spontaneous. The main binding forces in this interaction appear to be hydrophobic interactions.

## 3. Conclusion

In this study, the theoretically designed novel substituted tetrahydropyrimidine analogues (T_1–35_) were designed as promising DNA intercalators and Topo-II inhibitors. Notably, the theoretically designed analogue (T_30_) with a 3,4,5-trimethoxy phenyl side chain was found to be the superior candidate among the investigated compounds in comparison to both reference standards, Dox and EVP. Molecular dynamics simulation revealed that compound T_30_ showed the most stable behaviour with an L-RMSD value of 6.3 Å, as a maximum fluctuation level. However, Dox and EVP fluctuated to 8 and 9 Å, respectively, indicating their less stable behaviours compared to T_30_. Moreover, the target candidate (T_30_) achieved superior Δ*G* binding energy (−33.86 kcal mol^−1^), which was better than that of both Dox and EVP. Furthermore, the most promising candidate (T_30_) was synthesized and denoted as compound T_30_. Compound T_30_ revealed very strong antiproliferative activity with mean growth = −22% and mean GI% = 122%, and showed the greatest anticancer activity towards all 59 cell lines. We conclude that compound T_30_ is remarkably effective against non-small cell lung cancer (NCI–H522) and melanoma (LOX IMVI). Moreover, the binding interaction was investigated using several spectroscopic techniques, ionic strength studies, viscosity tests, and *in silico* investigations. The data demonstrated a strong affinity between compound T_30_ and ctDNA, indicating an intercalation binding relationship. Furthermore, the binding between compound T_30_ and ctDNA was spontaneous, and the resulting complex was mostly held together by hydrophobic interactions. In summary, this study presented comprehensive data on the characteristics of this interaction, including how the molecules connect, the strength of the binding, the specific location of the binding site, and the forces involved in the interaction. This information is highly significant for rationally designing anticancer candidates, intending to enhance their activity and selectivity.

## 4. Experimental

### 4.1. *In silico* studies

#### 4.1.1. Molecular docking

The theoretically designed substituted tetrahydropyrimidine analogues (T_1–35_) were subjected to a molecular docking process towards the hybrid DNA and Topo-II target receptor (PDB ID: 3QX3) to investigate their inhibitory potential. This was done using the MOE 2022.02 software,^68,69^ which was validated by redocking the co-crystallized EVP inhibitor inside its binding pocket. The obtained RMSD value (<2 Å) and the similar binding interactions confirmed the valid performance.^70^ All designed analogues (**T**_**1–35**_) were sketched in the working window of the MOE, and energy was minimized after partial charges optimization.^71^ Moreover, the target receptor (PDB ID: 3QX3) was downloaded and subjected to the preparation process by correction, 3D hydrogenation, and energy minimization steps.^72^ Then, a general docking process was carried out using a database containing the prepared designed analogues (**T**_**1–35**_), Dox, and EVP. Finally, the docked candidates were ranked according to their binding scores and RMSD values.

#### 4.1.2. Molecular dynamics simulation

The Desmond package of Schrödinger LLC^35^ was used to perform the molecular dynamics simulation at 500 ns^73^ for the superior designed analogue (T_30_), together with the two reference standards (Dox and EVP). The full compound methodology was described in the ESI data (ESI1).†

#### 4.1.3. MM-GBSA calculations

The thermal_mmgbsa.py python script of Schrödinger LLC was applied to calculate the Molecular Mechanics Generalized Born Surface Area (MM-GBSA) energies.^74^ The full compound methodology was described in the ESI data (SI2).†

### 4.2. Chemistry

The initial chemicals and reagents were acquired from Sigma-Aldrich and Merck Millipore, and were utilized without additional purification. The progression of the reactions was tracked using a pre-coated sheet of thin-layer chromatography obtained from Eastman Kodak Co. The mobile phase was a mixture of *n*-hexane and ethyl acetate at a ratio of 2 : 3. The melting point was determined using a Stuart SMP10 instrument and was not adjusted for any corrections. The PerkinElmer 2400 CHNS analyzer was used to conduct elemental analysis, and the results were found to be within ±0.40 of the theoretical values. 1D and 2D NMR spectra were recorded on a JEOL spectrometer using DMSO-*d*_6_. The HRMS were recorded on LC/Q-TOF, 6530 (Agilent Technologies, Santa Clara, CA, USA) at the Faculty of Pharmacy, Fayoum University. Compound T_30_ is 98.96% pure by HPLC analysis. The column used in HPLC was ZORBAX Eclipse plus C18 (150 × 4.6 mm, 3.5 μm). The mobile phase consisted of acetonitrile (ACN)/0.05 M KH_2_PO_4_ buffer pH 3.7 (30 : 70), with a flow rate of 1.5 mL min^−1^, and the run time was set as double the elution time. The ESI file† contains the spectrum data, as well as their interpretation.

#### 4.2.1. The preparation of ethyl 6-(chloromethyl)-1-methyl-2-oxo-4-(3,4,5-trimethoxyphenyl)-1,2,3,4-tetrahydropyrimidine-5-carboxylate (T_30_)

A mixture of ethyl 4-chloroacetoacetate 2 (4 mmol, 658 mg), 3,4,5-trimethoxybenzaldehyde 1 (2 mmol 392 mg), *N*-methyl urea 3 (4 mmol 412 mg), citric acid (2 mmol 384 mg), and TEOF (4 mmol 593 mg) was heated under reflux for 10 h in absolute ethanol. The reaction was stopped by adding 20 mL of distilled water, and the resulting liquid was subsequently chilled in an ice bath. The solid that formed was separated by filtration, and then purified through recrystallization using acetone. This process yielded the desired product, which is denoted as T_30_. Yield (370 mg, 93%) as yellow crystal with M.p. 245 °C. HPLC: *R*_T_ 8.21 min (purity: 98.96%). ^1^H NMR (500 MHz, DMSO-*d*_6_) *δ* (ppm): 1.11 (3H, t, *J* = 5.0 Hz, OCH_2_CH_3_), 3.17 (3H, s, NCH_3_), 3.57 (3H, s, 4-OCH_3_), 3.67 (6H, s, 3,5-diOCH_3_), 4.06 (2H, q, *J* = 5.0 Hz, OCH_2_CH_3_), 4.99 (1H, d, *J* = 10.0 Hz, CHCl), 5.08 (1H, d, *J* = 5.0 Hz, THPM 4-H), 5.11 (1H, d, *J* = 10.0 Hz, CHCl), 6.47 (2H, s, 2-H and 6-H), 8.06 (1H, d, *J* = 5.0 Hz, 3-NH). ^13^C NMR (126 MHz, DMSO) *δ* (ppm): 14.48 (OCH_2_CH_3_), 29.38 (NCH_3_), 38.49 (CH_2_Cl), 52.59 (4-C of THPM), 56.23 (3,5-diOCH_3_), 60.48 (4-OCH_3_), 60.92 (OCH_2_CH_3_), 103.59 (2-C and 6-C of benzene ring), 105.71 (5-C of THPM), 137.32 (1-C of benzene ring), 138.94 (4-C of benzene ring), 148.14 (6-C of THPM), 153.38 (3-C and 5-C of benzene ring), 153.56 (2-C of THPM), 164.98 (C=OOCH_2_). ^13^C NMR-DEPTQ spectrum, CH and CH_3_ [positive (up)], C_Q_ and CH_2_ [negative (down)], revealed the following signals at *δ*: 14.49 (OCH_2_CH_3_ ↑), 29.39 (NCH_3_ ↑), 38.50 (CH_2_Cl ↓), 52.59 (THPM 4-CH ↑), 56.24 (3,5-diOCH_3_ ↑), 60.48 (4-OCH_3_ ↑), 60.91 (OCH_2_CH_3_ ↓), 103.59 (2-CH and 6-CH of benzene ring ↑), 105.70 (5-C_Q_ of THPM ↓), 137.32 (1-C_Q_ of benzene ring ↓), 138.95 (4-C_Q_ of benzene ring ↓), 148.15 (6-C_Q_ of THPM ↓), 153.38 (3-C_Q_ and 5-C_Q_ of benzene ring ↓), 153.55 (C=O of THPM ↓), 164.99 (C=OOCH_2_ ↓). HRMS (ESI): *m/z*: Cald./Found 399.13174/399.13194 (C18H_24_^35^ClN_2_O_6_, [M + H]^+^), 401.12879/401.12784 (C18H_24_^37^ClN_2_O_6_, [M + H+2]^+^). Anal. Calcd. for C_18_H_23_ClN_2_O_6_: C, 54.21; H, 5.81; N, 7.02. Found: C, 53.99; H, 5.80; N, 6.97.

### 4.3. Biological evaluation

#### 4.3.1. *In vitro* antitumor screening against 59 cancer cell lines

The anticancer test was conducted on 59 human tumour cell lines derived from nine different tissues under the compound methodology of the Drug Evaluation Branch, National Cancer Institute, Bethesda, MD.^3,38,39^ All methods were carried out in accordance with relevant guidelines and regulations. The SRB assay protocol at 10 μM was employed and approved by the United States NCI to distinguish cell lethality from cell GI.

### 4.4. DNA-binding study

#### 4.4.1. Reagents and materials

Tris HCl, RB, EB, and ctDNA were obtained from Sigma Aldrich (St. Louis, MO, USA). A tris–HCl buffer solution (0.05 M) was made by dissolving 0.78 grams of tris–HCl in 100.0 mL of distilled water to get the desired final volume. The pH of the solution was adjusted to 7.4 using HCl solution (1 M). The ctDNA stock solution was obtained by dissolving 0.01 grams of ctDNA in Tris–HCl buffer, and bringing the total volume with the buffer to 50.0 mL. The solution was subsequently stored at a temperature of 4 °C, away from light, for a maximum duration of 5 days. Prior to conducting the studies, the ctDNA solution was subjected to sonication to confirm the uniformity of the solution. The ctDNA solution exhibited an absorbance ratio (*A*_260_/*A*_280_) of 1.92 (above 1.8),^67^ suggesting that the ctDNA was devoid of proteins. The concentration of the final ctDNA solution was determined using the extinction coefficient (6600 M^−1^ cm^−1^) of a single nucleotide at 260 nm (*T* = 298 K).^4,75^

A stock solution of compound T_30_ with a conc. of 1.0 × 10^−3^ M was obtained by dissolving it in a solution of DMSO/methanol. Additionally, RB with a concentration of 2.0 × 10^−3^ M and EB with a concentration of 1.2 × 10^−3^ M were prepared in ethanol, and stored in a dark environment at a temperature of 4 °C.

#### 4.4.2. UV spectrophotometric measurements

The UV spectra were obtained by varying the concentrations of compound T_30_ in the range of 0 to 40.0 μM, while maintaining a constant ctDNA concentration of 97.0 μM. The experiments were conducted at 3 various temperature settings (298, 303, and 313 K) to investigate the binding constants, and the temperature effect on the interaction between compound T_30_ and ctDNA. In addition, the investigation of ionic strength involved determining the values of absorbance of the ctDNA-compound T_30_ mixture. The mixture consisted of 97.0 μM of ctDNA, 2.0 μM of compound T_30_, and NaCl solution with concentrations ranging from 0 to 0.07 M. The measurements were taken at a temperature of 298 K.

#### 4.4.3. Competitive binding spectrofluorimetric studies

The spectra of various mixture solutions containing ctDNA (97.0 μM) and fluorescent probes, specifically RB (1.27 μM) and EB (7.0 μM), acting as groove and intercalation binding probes respectively, were obtained using an Agilent Technologies Cary Eclipse Fluorescence Spectrometer equipped with a Xenon flash lamp. The measurements were conducted both in the absence and presence of compound T_30_. The emission fluorescence spectra for EB and RB were measured at 603 nm and 576 nm, after being excited at 525 nm and 465 nm, respectively.

#### 4.4.4. Viscosity measurements

Oswald's viscometer was employed to measure viscosity at a constant temperature of 298 K. The concentration of ctDNA in the Tris–HCl buffer solution (pH = 7.4) was 97.0 μM, while the concentrations of compound T_30_ ranged from 0 to 40.0 μM. The flow times of the ctDNA solutions, both alone and when mixed with compound T_30_ solutions, were examined in triplicate utilizing a digital timer. The equation (*η* = (*t* –*t*_0_)/*t*_0_) was employed to compute the viscosity values. In this equation, (*t*) signifies the ctDNA solutions' flow time, and (*t*_0_) symbolizes the Tris–HCl buffer flow time.^67^ The data were used to compute the relative specific viscosity (*η*/*η*_0_)^1/3^, where *η* and *η*_0_ represent the specific viscosities of ctDNA with or without compound T_30_, respectively.

## Data availability

All data generated or analyzed during this study are included in this published article and its ESI material.†

## Author contributions

Conceptualization and supervision: Ahmed A. Al-Karmalawy; data curation and visualization: Haytham O. Tawfik, Ayman Abo Elmaaty, Galal Magdy, Aya Saad Radwan, Radwan Alnajjar, Moataz A. Shaldam, and Ahmed A. Al-Karmalawy; methodology: Haytham O. Tawfik, Galal Magdy, Aya Saad Radwan, Radwan Alnajjar, Moataz A. Shaldam, Salem Salman Almujri, Abdullah Yahya Abdullah Alzahrani, and Ahmed A. Al-Karmalawy; Writing–review & editing: Haytham O. Tawfik, Ayman Abo Elmaaty, Galal Magdy, Aya Saad Radwan, Radwan Alnajjar, Moataz A. Shaldam, Arwa Omar Al Khatib, Salem Salman Almujri, Abdullah Yahya Abdullah Alzahrani, Ahmed A. Al-Karmalawy. All authors revised and approved the final submitted version of the manuscript.

## Conflicts of interest

The authors declared no conflict of interest.

## Supplementary Material

RA-015-D5RA02179K-s001
