# Supplementary material for: Targeted synthesis of a trimethoxyphenyltetrahydropyrimidine analogue designed as a DNA intercalator: in silico, multi-spectroscopic, thermodynamic, and in vitro approaches
Source: RSC Adv. 2025 May 8;15(19):14946–65. doi: 10.1039/d5ra02179k (PMC12061050; doi:10.1039/d5ra02179k)
Supplement: RA-015-D5RA02179K-s001 [file RA-015-D5RA02179K-s001.pdf]

Supplementary Information (SI) for RSC Advances.
This journal is © The Royal Society of Chemistry 2025

Supporting Information

Targeted Synthesis of Trimethoxyphenyltetrahydropyrimidine Analogue Designed as DNA Intercalator; *In silico*, Multi-spectroscopic, Thermodynamic, and *In vitro* Approaches

Ahmed A. Al-Karmalawy[1,2,*], Ayman Abo Elmaaty[3,4], Galal Magdy[5,6], Aya Saad Radwan[2], Radwan Alnajjar[7], Moataz A. Shaldam[8], Arwa Omar Al Khatib[9], Salem Salman Almujri[10], Abdullah Yahya Abdullah Alzahrani[11], Haytham O. Tawfik[12,*]

[1] Department of Pharmaceutical Chemistry, College of Pharmacy, The University of Mashreq, Baghdad 10023, Iraq.
[2] Department of Pharmaceutical Chemistry, Faculty of Pharmacy, Horus University-Egypt, New Damietta 34518, Egypt.

[3] Medicinal Chemistry Department, Faculty of Pharmacy, Port Said University, Port Said 42526, Egypt.
[4] Medicinal Chemistry Department, Clinical Pharmacy Program, East Port said National University, Port Said 42526, Egypt.
[5] Pharmaceutical Analytical Chemistry Department, Faculty of Pharmacy, Kafrelsheikh University, Kafrelsheikh, 33511, Egypt
[6] Department of Pharmaceutical Analytical Chemistry, Faculty of Pharmacy, Mansoura National University, Gamasa, 7731168, Egypt
[7] CADD Unit, Faculty of Pharmacy, Libyan International Medical University, Benghazi 16063, Libya.
[8] Department of Pharmaceutical Chemistry, Faculty of Pharmacy, Kafrelsheikh University, Kafrelsheikh 33516, Egypt.
[9] Faculty of Pharmacy, Hourani Center for Applied Scientific Research, Al-Ahliyya Amman University, Amman, Jordan.
[10] Department of Pharmacology, College of Pharmacy, King Khalid University, Asir-Abha 61421, Saudi Arabia.
[11] Department of Chemistry, Faculty of Science and Arts, King Khalid University, Mohail Assir 61421, Saudi Arabia.

[12] Department of Pharmaceutical Chemistry, Faculty of Pharmacy, Tanta University, Tanta, 31527, Egypt.

*Corresponding authors:

Ahmed A. Al-Karmalawy; Email: akarmalawy@horus.edu.eg

Haytham O. Tawfik; Email: haytham.omar.mahmoud@pharm.tanta.edu.eg

Supporting Information

Table S1. Docking scores, RMSD, and binding interactions of the theoretically designed novel substituted tetrahydropyrimidine analogues (**T1-35**).

Compound	Score (Kcal/mol)	RMSD (Å)	Interactions	Distance (Å)
**T1**	-6.18	1.61	DC11/H-acceptor	3.18
**T2**	-6.15	1.55	DC11/H-acceptor<br>DA12/pi-H	2.95<br>3.56
**T3**	-6.13	2.00	GLN778/H-acceptor<br>DC11/pi-H	3.53<br>4.25
**T4**	-6.33	1.51	DA12/H-acceptor<br>MET782/pi-H	3.16<br>4.25
**T5**	-5.81	1.96	DC11/H-acceptor	2.95
**T6**	-5.93	1.24	MET782/pi-H	3.59
**T7**	-6.27	1.27	GLN778/H-acceptor	3.41
**T8**	-6.33	2.08	GLN778/H-acceptor<br>DC11/pi-H<br>DC11/pi-H	3.19<br>4.27<br>3.99
**T8**	-6.70	1.46	DA12/H-acceptor<br>MET782/pi-H	3.17<br>4.41
**T10**	-6.43	1.14	MET782/pi-H	4.18
**T11**	-6.74	1.59	DC11/H-acceptor	3.14
**T12**	-6.42	1.52	MET782/pi-H	4.15
**T13**	-6.43	1.66	PRO819/pi-H	3.88
**T14**	-6.67	1.44	DA12/H-acceptor<br>MET782/pi-H	3.10<br>3.88
**T15**	-6.59	1.79	MET782/pi-H	4.19
**T16**	-6.19	1.07	GLN778/H-acceptor<br>DC11/pi-H<br>DC11/pi-H	3.37<br>4.49<br>4.08
**T17**	-6.53	1.57	MET782/pi-H	4.58
**T18**	-6.41	1.43	MET782/pi-H	4.33
**T19**	-6.20	1.07	GLN 778/H-acceptor	2.93
**T20**	-6.77	1.73	DC11/H-acceptor	3.18
**T21**	-6.57	1.35	DC11/H-acceptor<br>MET782/pi-H	3.18<br>4.16
**T22**	-5.73	1.11	DC11/H-acceptor	3.05
**T23**	-6.29	1.28	DA12/H-acceptor	3.34
**T24**	-6.61	1.42	DA12/H-acceptor<br>MET782/pi-H	3.16<br>4.07
**T25**	-6.91	1.98	DC11/H-acceptor<br>MET782/pi-H	3.23<br>4.43
**T26**	-6.28	1.64	MET781/H-donor	3.95
**T27**	-6.09	1.84	DA/12/pi-H	3.71
**T28**	-6.26	1.25	GLN778/H-acceptor	2.93

**T29**	-6.47	1.63	MET782/pi-H	4.61
**T30**	-7.06	1.56	ALA817/H-donor<br>MET782/H-donor<br>DC11/H-acceptor<br>DA12/pi-H	3.35<br>4.07<br>3.24<br>3.76
**T31**	-6.10	1.69	SER818/pi-H<br>SER818/pi-H	4.92<br>4.35
**T32**	-6.21	1.64	DC11/H-acceptor<br>MET782/pi-H<br>MET782/pi-H	3.23<br>4.16<br>4.24
**T33**	-5.83	1.05	DA12/H-donor<br>GLN778/H-acceptor	3.40<br>3.48
**T34**	-6.38	2.06	DC11/H-acceptor<br>MET782/pi-H	3.23<br>4.49
**T35**	-6.07	1.17	DC11/H-acceptor	2.83
**Dox**	-7.44	1.58	GLN778/H-donor<br>DA12/H-acceptor	3.12<br>3.85
**Co-Cryst ligand (EVP)**	-7.45	1.57	MET782/H-acceptor<br>DA12/H-donor	2.94<br>2.96

Supporting Information

A

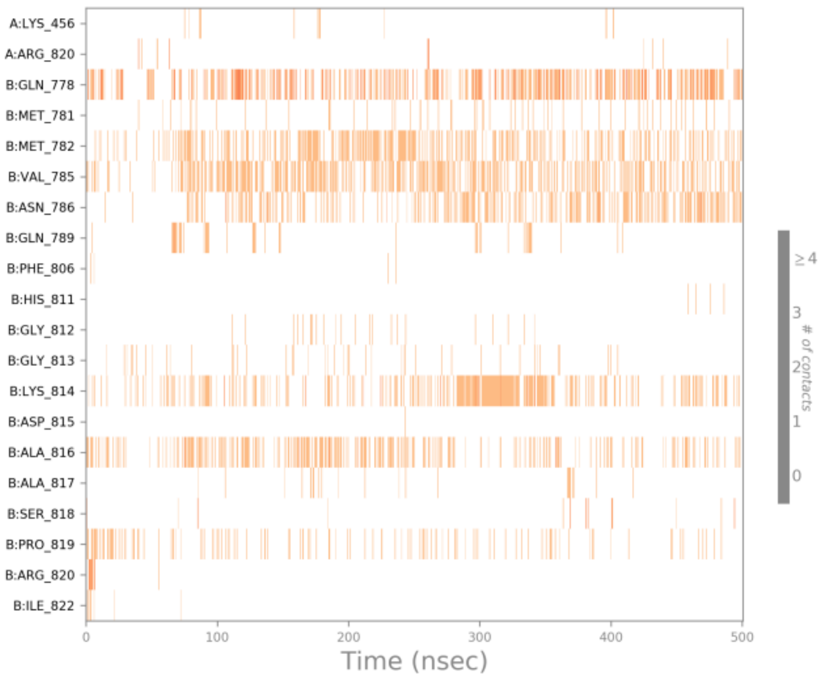

B

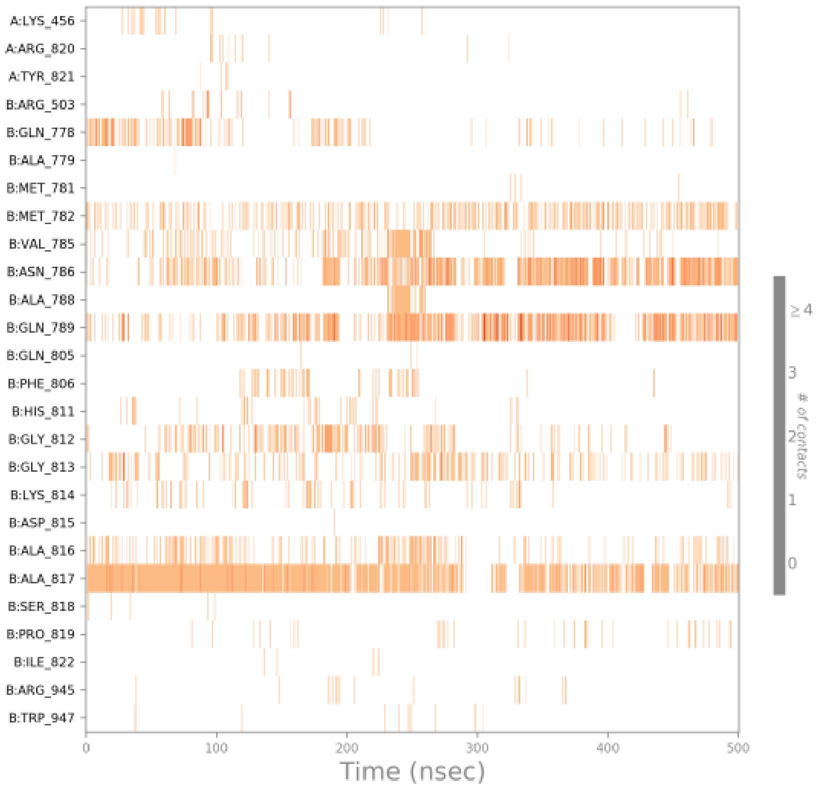

C

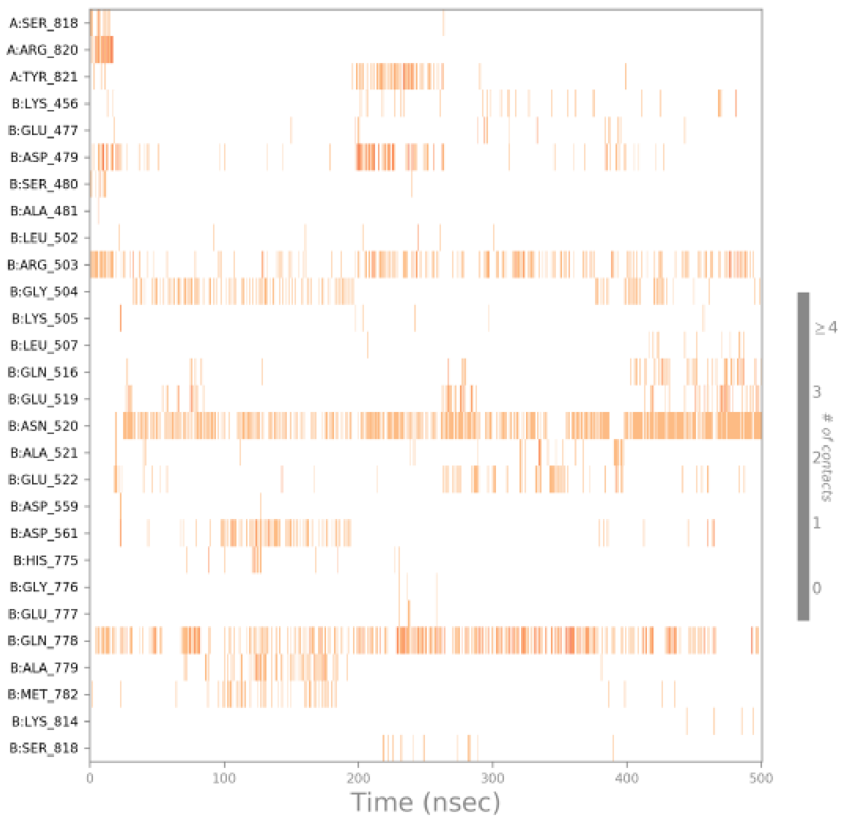

Figure S1. Heat map showing the total number of hybrid DNA and Topo-II target receptor-ligand interactions all over the simulation time of 500 ns for (**A**) **T_{30}**, (**B**) Dox, and (**C**) EVP.

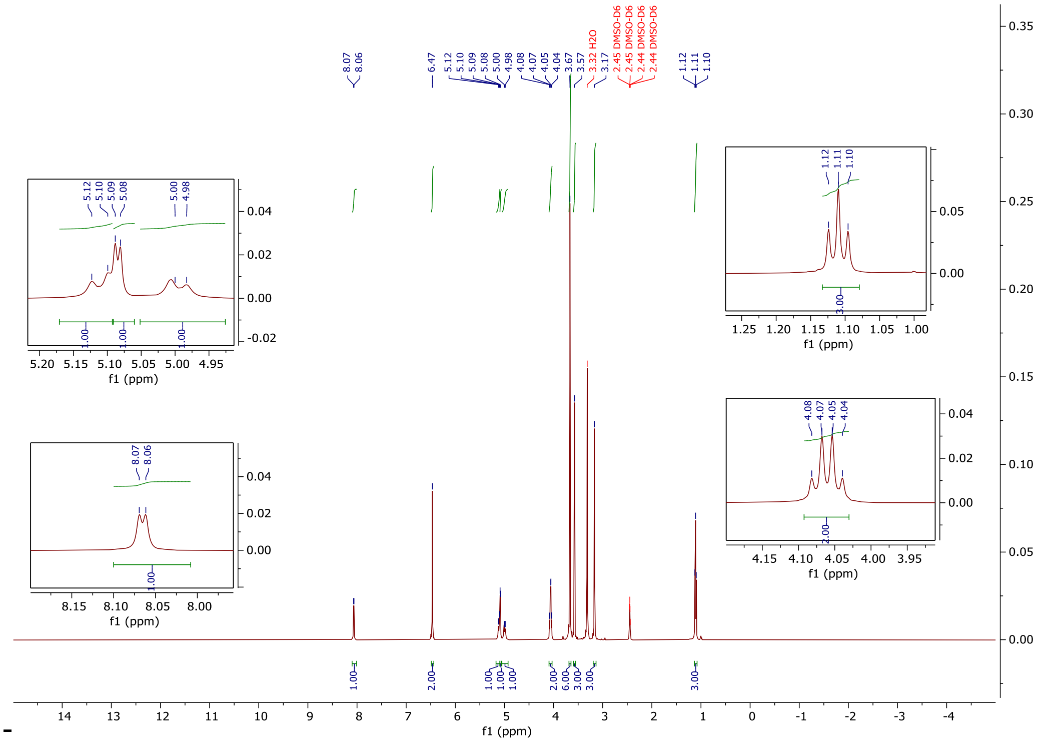

Figure S2. ^{1}H NMR spectrum (500 MHz, DMSO-d_6) of the compound $\mathbf{T_{30}}$.

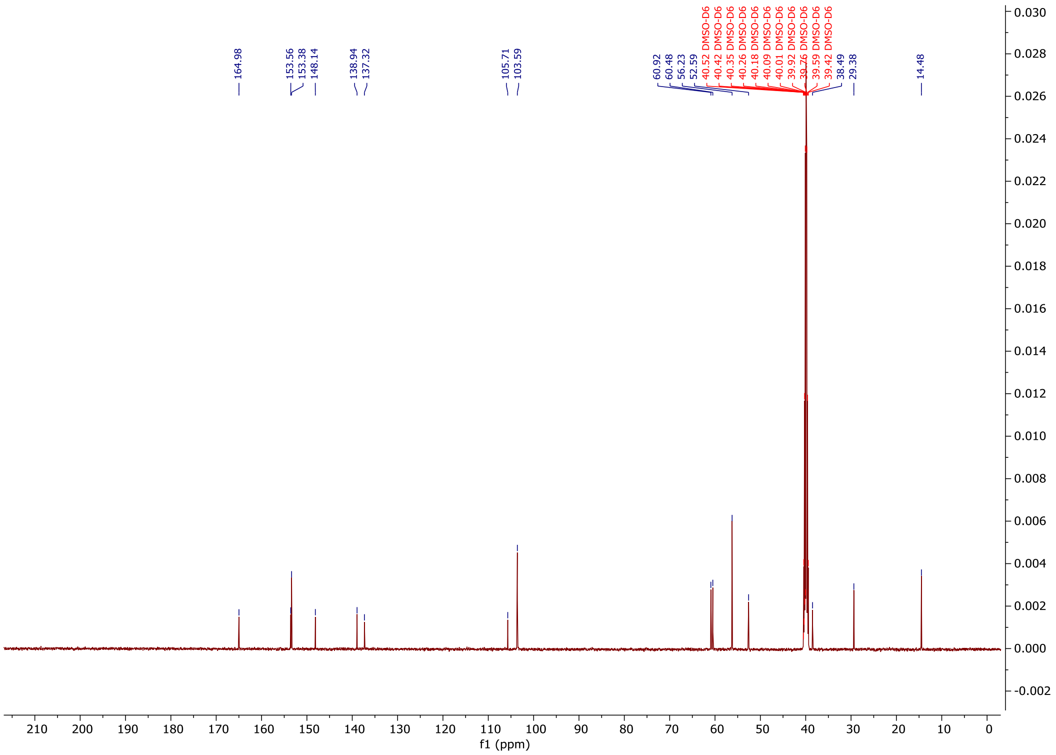

Figure S3. ^{13}C NMR spectrum (126 MHz, DMSO-d_6) of the compound $\mathbf{T_{30}}$.

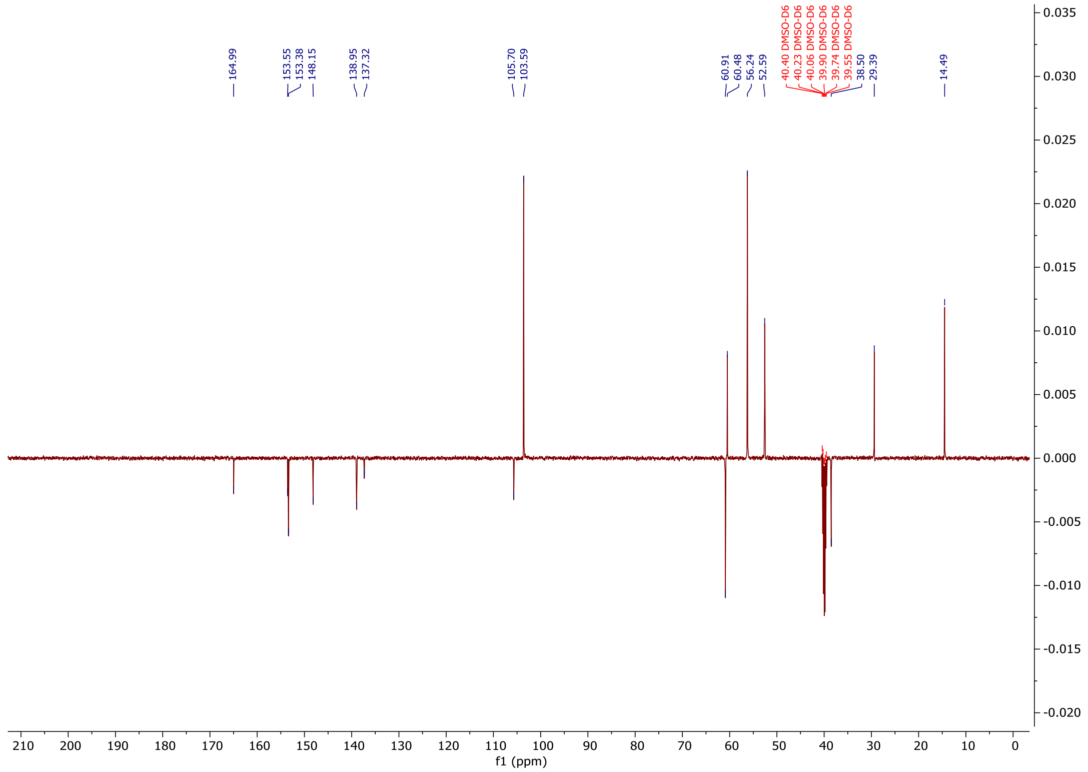

Figure S4. DEPTQ-^{13}C NMR spectrum (126 MHz, DMSO-d_6) of the compound $\mathbf{T_{30}}$.

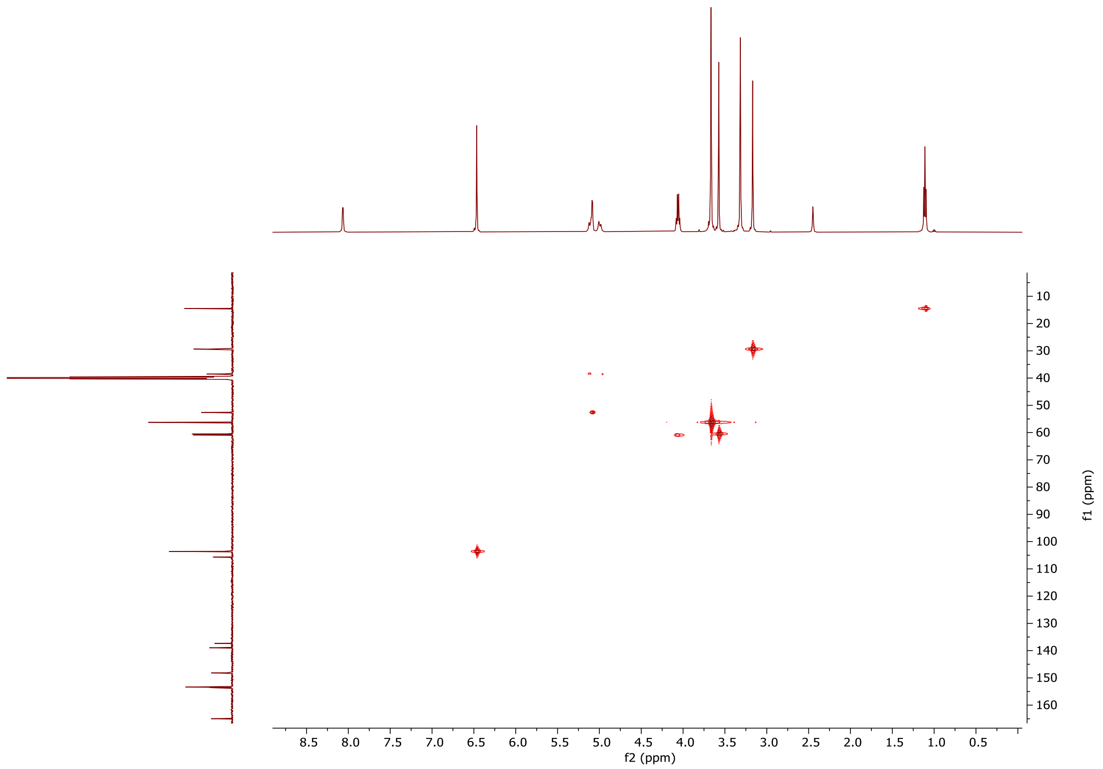

Figure S5. HMQC 2D spectrum (^{1}H NMR vs ^{13}C NMR) of the compound $\mathbf{T_{30}}$.

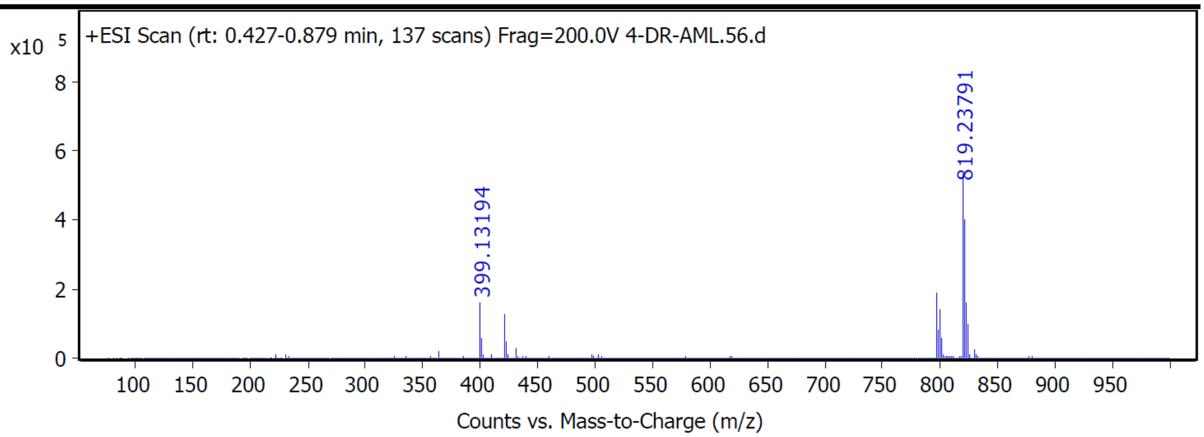

Figure S6. HRMS spectrum of the compound $\mathbf{T_{30}}$.

Acq. Operator : SYSTEM
Sample Operator : SYSTEM
Acq. Instrument : HPLC Location : Vial 10
Injection Date : 3/30/2024 1:51:59 PM
Inj Volume : 10.000 µl
Acq. Method : C:\CHEM32\1\METHODS\PURITY2022.M
Last changed : 3/30/2024 2:30:41 PM by SYSTEM
(modified after loading)
Analysis Method : C:\CHEM32\1\METHODS\DEF_LC.M14-6-22 water 40 ACN 60.M
Last changed : 3/30/2024 2:48:43 PM by SYSTEM
(modified after loading)
Sample Info : 60 ACN: 40 water, Flow 1.50 mL/min, 254 nm, 10 ul injection

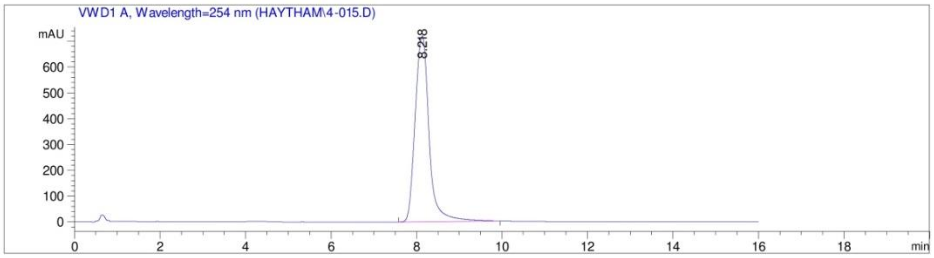

===
Area Percent Report
===

Sorted By : Signal
Calib. Data Modified : 3/30/2024 3:19:56 PM
Multiplier : 1.0000
Dilution : 1.0000
Sample Amount: : 0.10000 [ug/ml] (not used in calc.)
Do not use Multiplier & Dilution Factor with ISTDs

Signal 1: VWD1 A, Wavelength=254 nm

Peak #	RetTime [min]	Type	Width [min]	Area [mAU*s]	Area %	Name
1	8.218	BBA	0.2843	1.94358e4	98.9669	?
Totals :				1.94358e4	98.9669	

===
*** End of Report ***

Figure S7. HPLC spectrum of the compound $\mathbf{T_{30}}$.

Developmental Therapeutics Program One Dose Mean Graph	NSC: D-839923 / 1	Conc: 1.00E-5 Molar	Test Date: Nov 14, 2022
	Experiment ID: 2211OS69		Report Date: Jan 11, 2023

Panel/Cell Line	Growth Percent
**Leukemia**	
CCRF-CEM	11.67
HL-60(TB)	-21.68
K-562	2.36
MOLT-4	-27.73
RPMI-8226	11.00
SR	-22.25
**Non-Small Cell Lung Cancer**	
A549/ATCC	22.50
EKVX	-69.82
HOP-62	26.52
HOP-92	24.68
NCI-H226	-6.82
NCI-H23	-36.98
NCI-H322M	46.46
NCI-H460	9.62
NCI-H522	-76.95
**Colon Cancer**	
COLO 205	-96.59
HCC-2998	-20.75
HCT-116	-7.63
HCT-15	-64.13
HT29	-56.16
KM12	8.62
SW-620	-28.81
**CNS Cancer**	
SF-268	18.01
SF-295	14.59
SF-539	-57.59
SNB-19	34.67
SNB-75	-0.86
U251	-24.46
**Melanoma**	
LOX IMVI	-77.17
MALME-3M	-81.02
M14	-25.96
MDA-MB-435	9.09
SK-MEL-2	-32.94
SK-MEL-28	-37.94
SK-MEL-5	-84.42
UACC-257	-21.92
UACC-62	3.49
**Ovarian Cancer**	
IGROV1	-5.92
OVCAR-3	-90.15
OVCAR-4	-31.78
OVCAR-5	-54.72
OVCAR-8	5.35
NCI/ADR-RES	0.70
SK-OV-3	62.16
**Renal Cancer**	
786-0	-19.27
A498	20.37
ACHN	-83.70
CAKI-1	6.99
RXF 393	-67.31
TK-10	11.92
UO-31	-25.15
**Prostate Cancer**	
PC-3	23.99
DU-145	-87.43
**Breast Cancer**	
MCF7	-88.70
MDA-MB-231/ATCC	-31.53
HS 578T	-0.90
BT-549	-24.09
T-47D	-16.24
MDA-MB-468	-78.58
Mean	-22.23
Delta	74.36
Range	158.75

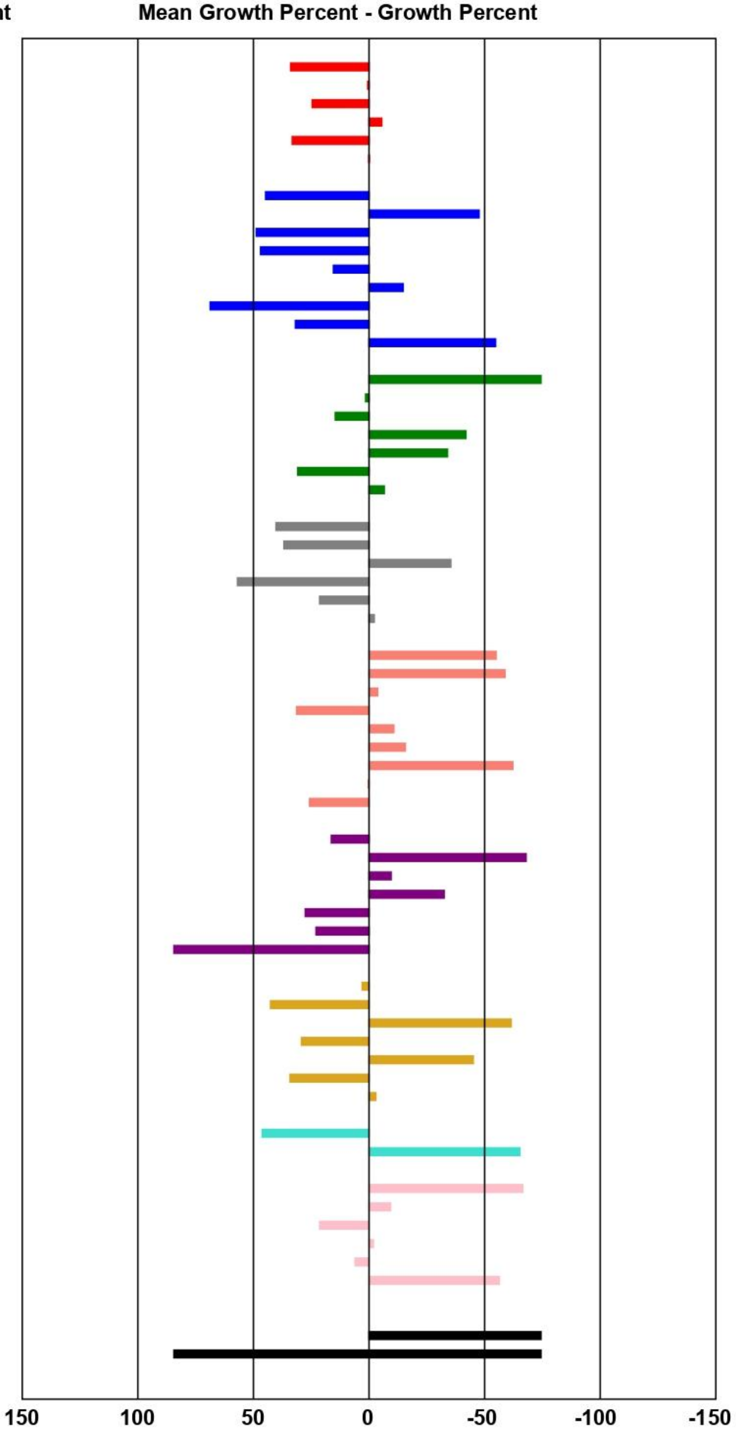

Figure S8. One dose mean graph for compound $\mathbf{T_{30}}$ (NSC 839923) at 10 µM.

National Cancer Institute Developmental Therapeutics Program In-Vitro Testing Results

NSC : D - 839923 / 1	Experiment ID : 2304NS09	Test Type : 08	Units : Molar
Report Date : October 16, 2023	Test Date : April 03, 2023	QNS :	MC :
COMI : 3OCH3_Cl	Stain Reagent : SRB Dual-Pass Related	SSPL : 1AYR	

Panel/Cell Line	Time Zero	Ctrl	Mean Optical Densities -8.0	-7.0	-6.0	-5.0	-4.0	Percent Growth -8.0	-7.0	-6.0	-5.0	-4.0	GI50	TGI	LC50
**Leukemia**															
CCRF-CEM	0.479	2.380	2.404	2.262	2.036	0.568	0.463	101	94	82	5	-3	2.59E-6	3.77E-5	> 1.00E-4
HL-60(TB)	0.992	3.087	3.071	2.932	2.833	0.851	0.738	99	93	88	-14	-26	2.35E-6	7.26E-6	> 1.00E-4
K-562	0.165	1.740	1.645	1.671	1.393	0.254	0.272	94	96	78	6	7	2.44E-6	> 1.00E-4	> 1.00E-4
MOLT-4	0.638	2.876	2.917	2.701	2.814	0.742	0.681	102	92	97	5	2	3.24E-6	> 1.00E-4	> 1.00E-4
RPMI-8226	1.038	2.987	2.912	2.821	2.688	0.932	1.020	96	91	85	-10	-2	2.32E-6	7.80E-6	> 1.00E-4
**Non-Small Cell Lung Cancer**															
A549/ATCC	0.381	2.494	2.426	2.389	2.313	1.242	0.122	97	95	91	41	-68	6.57E-6	2.37E-5	6.83E-5
EKVX	0.712	1.733	1.787	1.757	1.646	0.925	0.048	105	102	91	21	-93	3.87E-6	1.52E-5	4.18E-5
HOP-62	0.651	2.170	2.085	2.112	2.040	1.641	0.140	94	96	91	65	-79	1.27E-5	2.84E-5	6.33E-5
HOP-92	1.626	2.446	2.433	2.328	2.344	1.797	0.364	98	86	88	21	-78	3.66E-6	1.63E-5	5.24E-5
NCI-H226	0.748	2.155	2.107	2.059	2.074	1.177	0.177	97	93	94	30	-76	4.94E-6	1.93E-5	5.66E-5
NCI-H23	0.689	1.983	1.921	1.883	1.819	0.753	0.059	95	92	87	5	-92	2.84E-6	1.12E-5	3.71E-5
NCI-H322M	0.905	2.345	2.283	2.239	2.218	1.655	0.022	96	93	91	52	-98	1.03E-5	2.23E-5	4.81E-5
NCI-H460	0.251	2.783	3.067	3.097	2.829	0.673	0.055	111	112	102	17	-78	4.06E-6	1.50E-5	5.05E-5
NCI-H522	0.929	2.127	2.013	1.938	1.872	0.262	0.272	90	84	79	-72	-71	1.55E-6	3.33E-6	7.16E-6
**Colon Cancer**															
COLO 205	0.402	1.837	1.888	1.842	1.907	0.527	0.118	104	100	105	9	-71	3.72E-6	1.29E-5	5.49E-5
HCC-2998	0.766	2.515	2.373	2.303	2.437	1.219	0.054	92	88	96	26	-93	4.51E-6	1.65E-5	4.35E-5
HCT-116	0.379	3.025	2.911	3.008	2.929	0.569	0.295	96	99	96	7	-22	3.31E-6	1.76E-5	> 1.00E-4
HCT-15	0.224	1.848	1.850	1.658	1.626	0.071	0.015	100	88	86	-69	-93	1.72E-6	3.61E-6	7.59E-6
HT29	0.231	1.769	1.762	1.754	1.702	0.194	0.139	99	99	96	-16	-40	2.56E-6	7.16E-6	> 1.00E-4
KM12	0.593	2.862	2.938	2.986	2.940	1.178	0.032	103	105	103	26	-95	4.87E-6	1.64E-5	4.26E-5
SW-620	0.316	2.228	2.242	2.371	2.311	0.238	0.050	101	107	104	-25	-84	2.64E-6	6.44E-6	2.66E-5
**CNS Cancer**															
SF-268	0.591	2.008	2.167	2.078	1.916	0.840	0.140	111	105	94	18	-76	3.74E-6	1.54E-5	5.24E-5
SF-295	0.706	2.733	2.724	2.724	2.814	1.631	0.049	100	100	104	46	-93	8.42E-6	2.13E-5	4.89E-5
SF-539	0.766	2.244	2.229	2.261	2.151	0.731	0.003	99	101	94	-5	-100	2.78E-6	8.98E-6	3.00E-5
SNB-19	0.698	2.040	1.917	1.910	1.952	1.451	0.002	91	90	93	56	-100	1.09E-5	2.29E-5	4.79E-5
SNB-75	1.302	2.389	2.344	2.409	2.213	1.607	0.069	96	102	84	28	-95	4.03E-6	1.69E-5	4.32E-5
U251	0.358	1.905	1.815	1.836	1.728	0.705	0.072	94	96	89	22	-80	3.83E-6	1.66E-5	5.10E-5
**Melanoma**															
LOX IMVI	0.194	1.387	1.242	1.251	1.044	0.065	0.009	88	89	71	-66	-95	1.43E-6	3.29E-6	7.59E-6
MALME-3M	0.550	1.832	1.722	1.639	1.510	0.266	0.049	91	85	75	-52	-91	1.57E-6	3.91E-6	9.71E-6
M14	0.556	2.447	2.419	2.389	2.375	0.931	0.280	99	97	96	20	-50	4.02E-6	1.93E-5	> 1.00E-4
MDA-MB-435	0.519	2.545	2.440	2.359	2.304	0.554	0.045	95	91	88	2	-91	2.76E-6	1.04E-5	3.59E-5
SK-MEL-2	0.954	2.504	2.458	2.374	2.419	1.260	0.237	97	92	94	20	-75	3.94E-6	1.61E-5	5.43E-5
SK-MEL-28	0.555	1.832	1.825	1.783	1.751	0.610	0.012	99	96	94	4	-98	3.08E-6	1.10E-5	3.40E-5
SK-MEL-5	0.759	3.155	3.124	3.103	3.007	0.905	0.072	99	98	94	6	-91	3.16E-6	1.16E-5	3.80E-5
UACC-257	0.920	2.409	2.272	2.274	2.116	1.239	0.128	91	91	80	21	-86	3.27E-6	1.58E-5	4.61E-5
UACC-62	0.916	2.728	2.586	2.522	2.381	0.788	0.012	92	89	81	-14	-99	2.11E-6	7.11E-6	2.66E-5
**Ovarian Cancer**															
IGROV1	0.632	2.527	2.567	2.468	2.447	0.964	0.081	102	97	96	17	-87	3.84E-6	1.47E-5	4.41E-5
OVCAR-3	0.572	1.883	1.957	2.321	1.958	0.483	0.010	106	133	106	-16	-98	2.88E-6	7.43E-6	2.61E-5
OVCAR-4	0.977	2.375	2.367	2.388	2.297	1.587	0.014	99	101	94	44	-99	7.49E-6	2.03E-5	4.55E-5
OVCAR-5	0.522	1.654	1.596	1.557	1.523	0.669	0.006	95	91	88	13	-99	3.23E-6	1.31E-5	3.66E-5
OVCAR-8	0.514	2.385	2.328	2.309	2.338	1.132	0.100	97	96	97	33	-81	5.45E-6	1.95E-5	5.37E-5
NCI/ADR-RES	0.536	1.907	1.902	1.886	1.832	0.830	0.154	100	98	95	21	-71	4.07E-6	1.70E-5	5.89E-5
SK-OV-3	0.770	1.868	1.842	1.777	1.830	1.552	0.077	98	92	97	71	-90	1.35E-5	2.77E-5	5.65E-5
**Renal Cancer**															
786-0	0.598	2.684	2.545	2.646	2.629	0.959	0.244	93	98	97	17	-59	3.90E-6	1.68E-5	7.58E-5
A498	1.211	2.157	2.174	2.072	2.037	1.625	0.043	102	91	87	44	-96	7.20E-6	2.05E-5	4.66E-5
ACHN	0.387	1.716	1.690	1.688	1.478	0.392	-0.001	98	98	82	0	-100	2.47E-6	1.01E-5	3.17E-5
CAKI-1	0.422	1.747	1.648	1.585	1.346	0.450	0.007	93	88	70	2	-98	1.96E-6	1.05E-5	3.30E-5
RXF 393	1.092	1.830	1.808	1.812	1.710	1.019	0.276	97	97	84	-7	-75	2.36E-6	8.42E-6	4.33E-5
SN12C	0.584	2.235	2.176	2.139	2.105	0.835	0.007	96	94	92	15	-99	3.53E-6	1.36E-5	3.73E-5
TK-10	1.023	2.205	2.013	1.974	1.904	1.072	0.078	84	80	75	4	-92	2.23E-6	1.10E-5	3.64E-5
UO-31	0.850	2.171	1.906	1.900	1.763	0.788	0.029	80	79	69	-7	-97	1.78E-6	8.01E-6	3.00E-5
**Prostate Cancer**															
PC-3	0.726	2.696	2.595	2.661	2.713	1.624	0.287	95	98	101	46	-60	8.32E-6	2.69E-5	7.97E-5
DU-145	0.246	1.140	1.115	1.131	0.885	0.323	0.012	97	99	71	9	-95	2.20E-6	1.21E-5	3.66E-5
**Breast Cancer**															
MCF7	0.428	2.430	2.244	2.259	1.992	0.472	0.098	91	91	78	2	-77	2.34E-6	1.07E-5	4.54E-5
MDA-MB-231/ATCC	0.842	2.346	2.364	2.320	2.249	0.892	0.021	101	98	94	3	-98	3.04E-6	1.08E-5	3.38E-5
HS 578T	1.190	2.174	2.159	2.134	2.065	1.122	0.771	98	96	89	-6	-35	2.58E-6	8.70E-6	> 1.00E-4
BT-549	1.278	2.369	2.357	2.363	2.325	1.383	0.316	99	99	96	10	-75	3.40E-6	1.30E-5	5.04E-5
T-47D	0.723	1.914	1.914	1.833	1.696	0.930	0.457	100	93	82	17	-37	3.11E-6	2.09E-5	> 1.00E-4
MDA-MB-468	0.843	2.070	2.102	2.011	1.841	0.890	0.288	103	95	81	4	-66	2.53E-6	1.13E-5	5.91E-5

(Log10 Concentration applies to the Mean Optical Densities and Percent Growth columns.)

Figure S9. Values of log molar concentration of response parameters ($\log_{10}$ GI_{50}, $\log_{10}$ TGI & $\log_{10}$ LC_{50}) for compound $\mathbf{T_{30}}$.

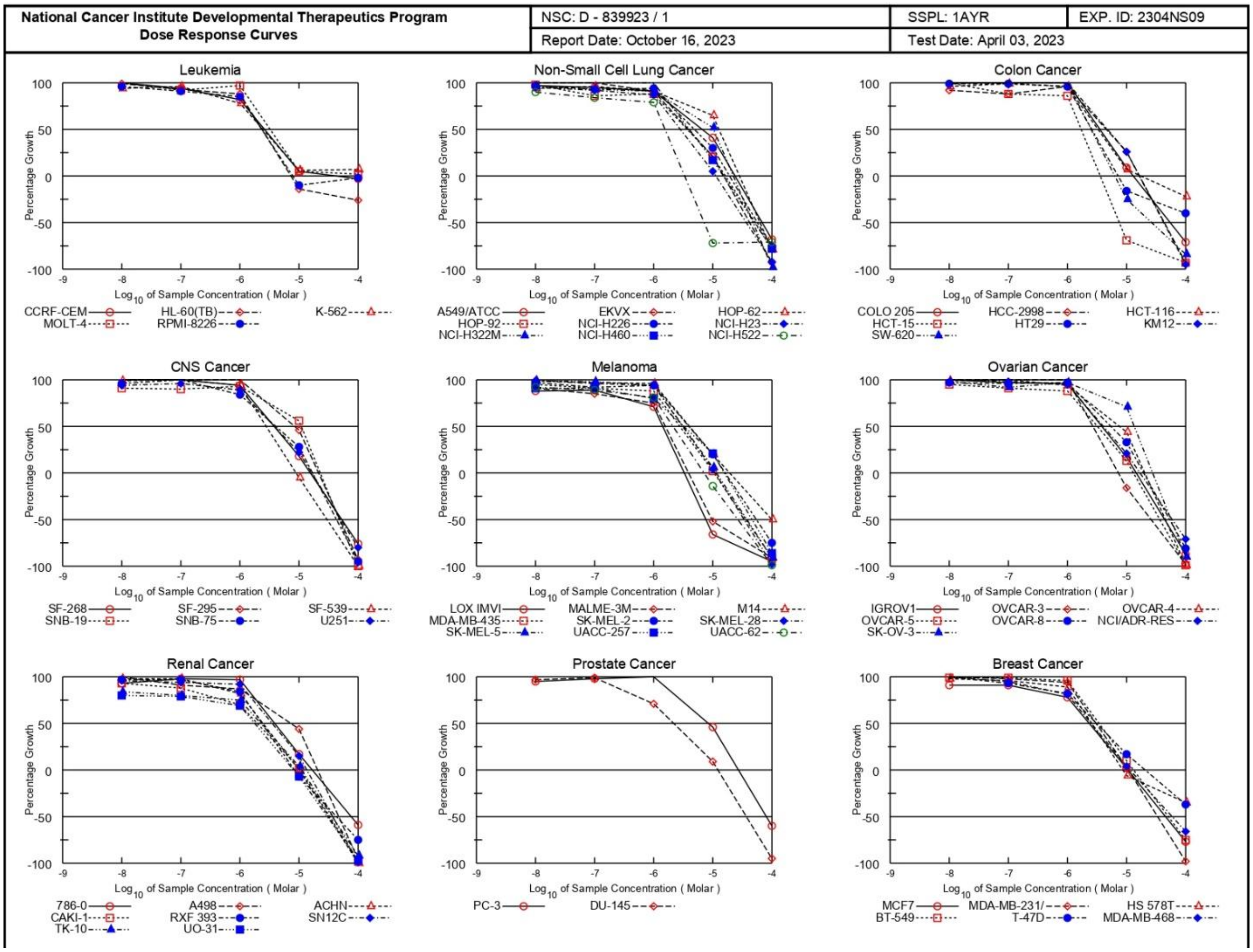

Figure S10. Dose-response curves (% growth versus sample concentration) for all cell lines with different subpanel obtained from the NCI's *in vitro* disease-oriented human cancer cells line for compound $\mathbf{T_{30}}$ on nine types of cancer.

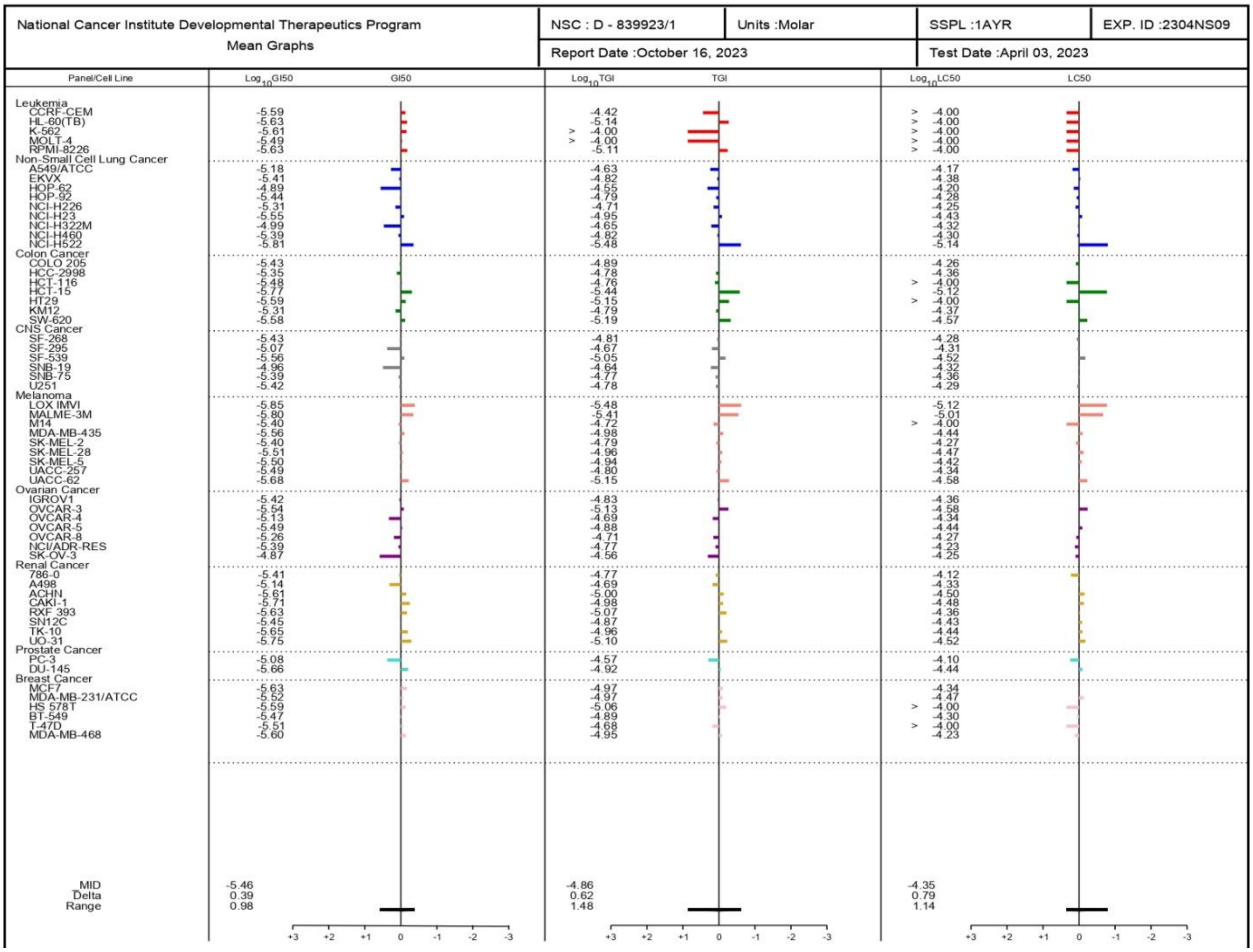

Figure S11. Mean Graphs of the $\log_{10}$ values (Molar) of GI_{50}, TGI, and LC_{50} obtained from the NCI 60 cell line experiments for compound $\mathbf{T_{30}}$.

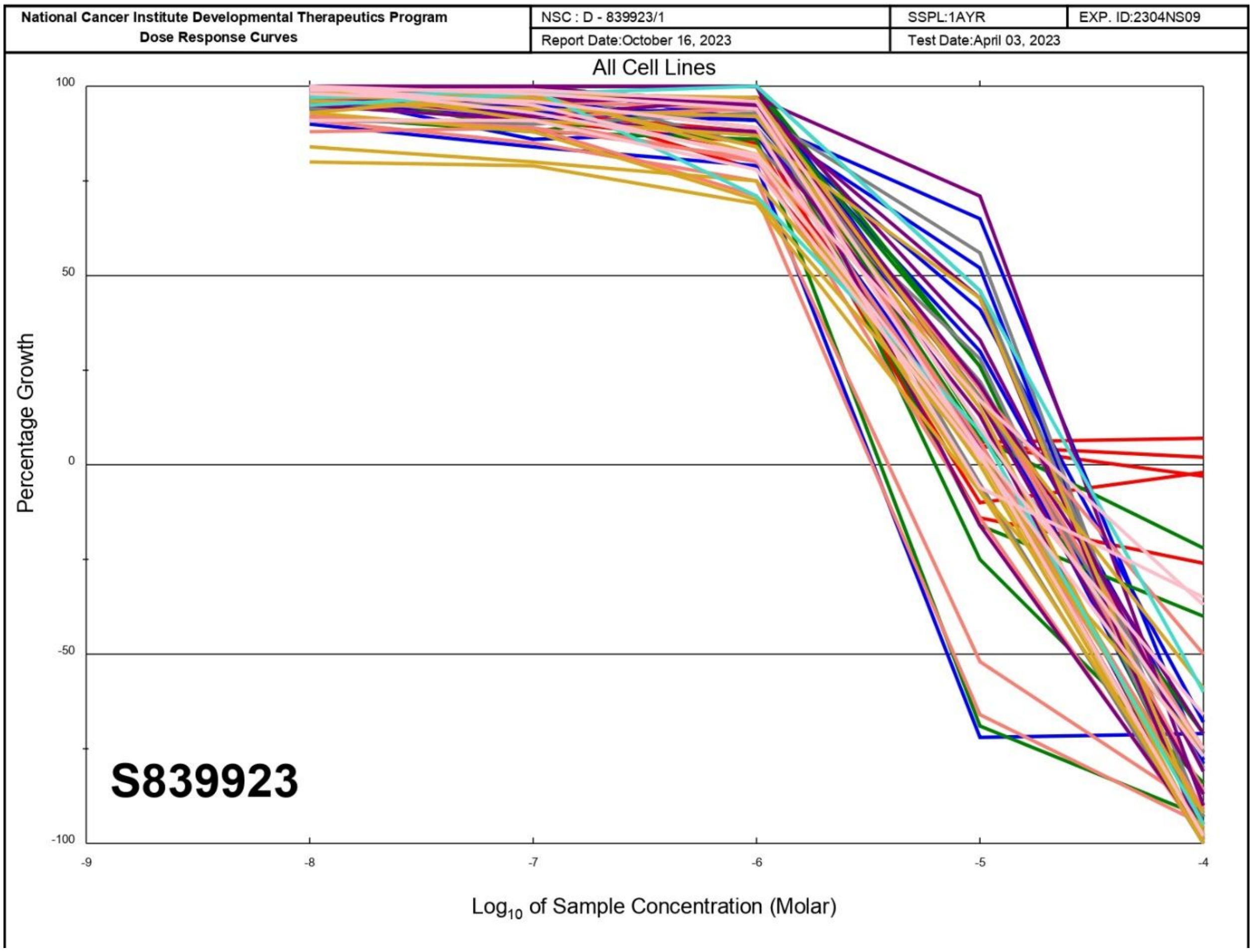

Figure S12. Dose-response curves for all cell lines in the NCI 60 panel exposed compound $\mathbf{T_{30}}$ with tissue-originated colors and shapes.

Supporting Information

SI1. Molecular dynamics simulations

The molecular dynamics simulations were carried out using the Desmond simulation package of Schrödinger LLC.[1-3] The NPγT ensemble with the temperature 300 K and a pressure 1.01 bar was applied in all runs. The simulation length was 500 ns with a relaxation time of 1 ps. The OPLS4 force field parameters were used in all simulations.[4] The cutoff radius in Coulomb interactions was 9.0 Å. The orthorhombic periodic box boundaries were set 10 Å away from the protein atoms. The water molecules were explicitly described using the transferable intermolecular potential with the three points (TIP3P) model.[5] Salt concentration was set to 0.15 M NaCl and was built using the System Builder utility of Desmond. The Martyna−Tuckerman−Klein chain coupling scheme with a coupling constant of 2.0 ps was used for the pressure control and the Nosé−Hoover chain coupling scheme for the temperature control.[6, 7] Nonbonded forces were calculated using a RESPA integrator where the short-range forces were updated every step, and the long-range forces were updated every three steps. The trajectories were saved at 300 ps intervals for analysis. The behavior and interactions between the ligands and protein were analyzed using the Simulation Interaction Diagram tool implemented in the Desmond MD package. The stability of MD simulations was monitored by looking at the RMSD of the ligand and protein atom positions as a function of simulation time.

SI2. MD trajectory analysis and prime MM-GBSA calculations

Simulation interactions diagram panel of Maestro software was used to monitoring interactions contribution in the ligand-protein stability. The molecular mechanics generalized born/solvent accessibility (MM – GBSA) was performed to calculate the ligand binding free energies and ligand strain energies for docked compounds over the last 50 ns with thermal_mmgbsa.py python script provided by Schrodinger which takes a Desmond trajectory file, splits it into individual snapshots, runs the MM-GBSA calculations on each frame, and outputs the average computed binding energy.

References

1. K. J. Bowers, D. E. Chow, H. Xu, R. O. Dror, M. P. Eastwood, B. A. Gregersen, J. L. Klepeis, I. Kolossvary, M. A. Moraes, F. D. Sacerdoti, J. K. Salmon, Y. Shan and D. E. Shaw, 2006.
2. M. H. El-Shershaby, A. Ghiaty, A. H. Bayoumi, A. A. Al-Karmalawy, E. M. Husseiny, M. S. El-Zoghbi and H. S. Abulkhair, *Bioorganic & Medicinal Chemistry*, 2021, **42**, 116266.
3. D. E. S. Research, *Journal*, 2021.
4. E. Harder, W. Damm, J. Maple, C. Wu, M. Reboul, J. Y. Xiang, L. Wang, D. Lupyan, M. K. Dahlgren, J. L. Knight, J. W. Kaus, D. S. Cerutti, G. Krilov, W. L. Jorgensen, R. Abel and R. A. Friesner, *Journal of Chemical Theory and Computation*, 2016, **12**, 281-296.
5. W. L. Jorgensen, J. Chandrasekhar, J. D. Madura, R. W. Impey and M. L. Klein, *Journal of Chemical Physics*, 1983, **79**, 926-935.
6. G. J. Martyna, M. L. Klein and M. Tuckerman, *Journal of Chemical Physics*, 1992, **97**, 2635-2643.
7. G. J. Martyna, D. J. Tobias and M. L. Klein, *Journal of Chemical Physics*, 1994, **101**, 4177-4189.
